# Emission behaviors of unsymmetrical 1,3-diaryl-β-diketones: A model perfectly disclosing the effect of molecular conformation on luminescence of organic solids

**DOI:** 10.1038/srep09140

**Published:** 2015-03-16

**Authors:** Xiao Cheng, Feng Li, Shenghua Han, Yufei Zhang, Chuanjun Jiao, Jinbei Wei, Kaiqi Ye, Yue Wang, Hongyu Zhang

**Affiliations:** 1 State Key Laboratory of Supramolecular Structure and Materials, College of Chemistry, Jilin University, 2699 Qianjin Avenue, Changchun 130012, P. R. China

## Abstract

A series of unsymmetrical 1,3-diaryl-β-diketones 1–6 displaying molecular conformation-dependent fluorescence quantum yields have been synthesized. Crystals with planar molecular conformation such as 1, 2, 3 and 4 are highly fluorescent (*φ*_f_: 39–53%), and the one holding slightly twisted conformation (5) is moderately luminescent (*φ*_f_ = 17%), while crystal 6 possessing heavily bent structure is completely nonluminous (*φ*_f_ ~ 0). The distinct fluorescence efficiencies are ascribed to their different molecular conformations, since all the crystals hold the same crystal system, space group and crystal packing structures. Additionally, the fluorescent crystals 1–5 display low threshold amplified spontaneous emission (ASE) with small full widths at half-maximum (FWHM: 3–7 nm), indicating their potential as candidates for organic crystal lasing devices.

Luminescent π-conjugated organic solids have wide applications in optoelectronics such as organic light-emitting diodes (OLEDs) and organic solid-state lasers (OSLs)^1^,^2^,^3^,^4^,^5^,^6^,^7^,^8^,^9^,^10^,^11^,^12^,^13^,^14^,^15^. To achieve ideal device performance, the emission color and efficiency of the luminescent organic materials should be optimized. How to design and synthesize organic materials with desirable emission color and high efficiency has been a challenging task in material science during the past decades. In the initial stage of exploring high-performance luminescent organic materials, the optimization of molecular structure is mainly concerned since the luminescent properties of organic solids are considered to be determined by the chemical structure of the constituent molecule^16^,^17^,^18^,^19^. Recently, it has been realized that material morphology optimization is also important in terms of device performance^20^,^21^. The emission color or quantum yield of organic luminescent materials in the solid states is decided by not only the chemical structure of the constituent molecules, but also the molecular conformation or the intermolecular interactions between neighboring molecules^22^,^23^,^24^,^25^,^26^,^27^,^28^,^29^,^30^. In this sense, it is more reasonable to optimize the structure of material at both molecular and supramolecular levels for constructing high-performance optoelectronic devices. Thus, the structure-property relationship appears seasonably as an important issue in material science and has attracted great attentions in recent years. The deep understanding and comprehensive consideration of this relationship at both molecular structure and supramolecular structure (a higher level of “structure”) levels may provide valuable information in guiding the design and synthesis of high performance organic optoelectronic material.

Rigid π-fused skeletons such as acene are ideal candidates to illustrate the effect of molecular packing on emission properties including both color and efficiency^31^,^32^,^33^. Generally, the emission of the π-conjugated molecules would be red-shifted with reduced quantum yield when the molecules largely stack in aggregated states. π-Conjugated systems with relatively flexible framework such as oligo(phenylene vinylene) have been chosen to reveal the impact of conformation on emission^34^,^35^. Indeed, plenty of molecules have been constructed regarding the structure-property relationship^36^,^37^,^38^,^39^. Some important conclusions have been drawn, for instance, J-aggregation is beneficial for the improvement of fluorescence efficiency while H-aggregation shows negative effect^39^. Nevertheless, emphasis is mainly focused on the dependence of the emission color on the packing mode. The reported examples disclosing the molecular conformation on luminescent properties usually involve contribution from molecular packing effects. The model system that will give a clear picture of how molecular conformation affects emission property is still very rare.

Our group is continuously interested in designing simple molecular systems that can precisely disclose the influence of molecular conformation or packing on luminescent properties of organic materials. For instance, we designed an anthracene-containing molecule which could form five polymorphs with quite different emission colors associated to anthracene packing modes and thus definitely expounded molecular arrangement effects on emission color^31^. Another molecular system of aromatic-amine with relatively flexible framework has been designed in our lab. These molecules displayed dramatically different emission colors and provided the combined effect of molecular conformation and packing on fluorescence colors^40^. Very recently, we employed a semi-rigid molecule to construct organic polymorphs that exhibited the individual effect of molecular conformation and arrangement on emission colors for the first time^41^. Most of the examples reported so far in this area are related to emission colors; and the fluorescence efficiency has not been deeply investigated, especially, the unadulterated relationship between molecular conformation and efficiency of luminescent materials has never been revealed. The difficulty lies on that the molecular conformation variation usually induces molecular packing mode change. Therefore, it is hard to distinguish the impact degree of conformation and arrangement on luminescent properties.

We herein provided a very simple molecular system of 1,3-diaryl-β-diketones **1**–**6** (Figure 1) that perfectly disclosed the relationship between molecular conformation and fluorescence efficiency of organic solids. The high crystalline nature of these linear molecules as well as the intrinsic intra/inter molecular hydrogen bonds allowed the readily formation of high quality crystals for all the compounds. The as-prepared crystalline samples exhibited totally different quantum yields which were merely associated to their molecular conformations on the basis of photophysical data, crystallography analysis and theoretical calculations. In this contribution, we mainly focus on the viewpoint of how molecular conformation affects the fluorescence efficiency based on these simple molecular fluorophores. The excellent amplified spontaneous emissions (ASE) of the fluorescent crystals **1**–**5**, which strongly implies the potential application of these crystals in OSLs will be also presented.

## Results and Discussion

### Synthesis and crystal growth

Compounds **1** and **5** have been previously reported as reaction intermediates and **2**, **3**, **4**, and **6** are novel molecules which are newly synthesized in this work according to the known procedure^42^,^43^. A mixture of ethyl-4-(dimethylamino)benzoate and 2′-hydroxyacetophenone derivatives was heated at 60°C for 16 hours in the presence of sodium hydride. The reaction mixture was poured into icy hydrochloric acid (0.1 M) and the precipitate was filtered. Purification of the precipitate by simple crystallization from CHCl_3_/CH_3_OH (1:2) gave rise to the desired product as bulky crystals with the length of about 1–2 mm in high yields 52–64%. The crystals are characterized by NMR, element analysis and mass spectrometry. All the compounds are soluble in common organic solvents such as dichloromethane (CH_2_Cl_2_), chloroform (CHCl_3_) and tetrahydrofuran (THF) etc. NMR spectra of each molecule contain two sets of signals, reflecting both the enol and keto forms can stably exist in deuterated chloroform. The integration values of the hydroxyl protons indicate the enol form is more stable than the keto form in solution. Due to the high crystalline nature of these molecules, the purification of the products allows the synthesis of high quality crystals which are suitable for X-ray diffraction measurement. It is worthy to note that all the compounds crystallize into the very similar flake-like crystals with comparable size and thickness, demonstrating the crystallization behavior of these molecules is dominantly determined by the conjugated 1,3-diaryl-β-diketone backbone. Crystals **2**, **3**, **4** and **6** hold the same rhombus shape, crystal **1** is rectangular, and crystal **5** displays an interesting shuttle shape. The shape of the organic crystals obtained by solution approaches may be affected by solvent polarity, solvent composition, concentration, crystallizing temperature and surfactant etc. Since the crystallizing conditions mentioned above for compounds **1**–**6** are quite similar, the difference in crystal shape for these compounds is considered to originate mainly from the substituents.

### Optical properties in various states

**Figure 2:**
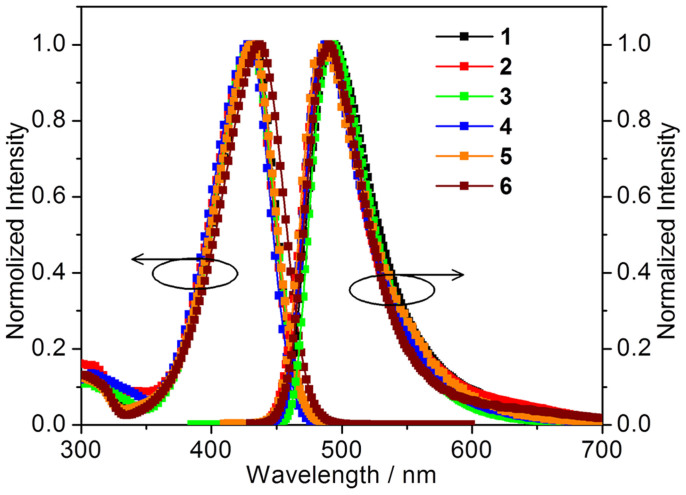
Absorption and emission spectra of compounds 1–6 in CH_2_Cl_2_ (1 × 10^−5^ M).

**Figure 3:**
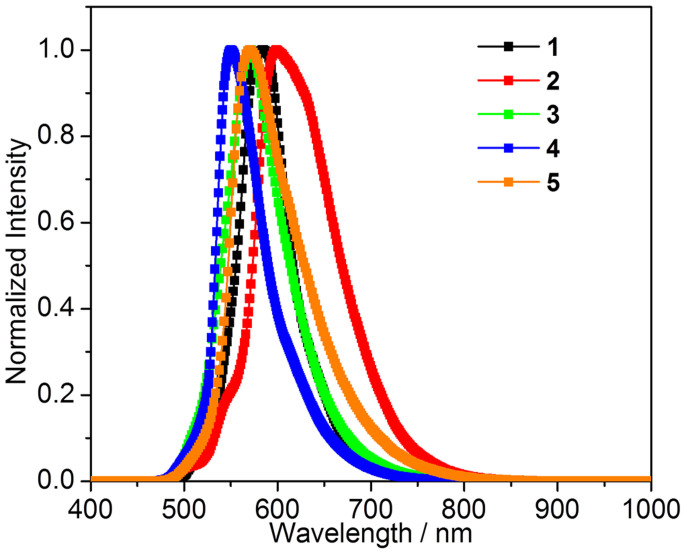
Emission spectra of bulky crystals 1–6.

**Figure 4:**
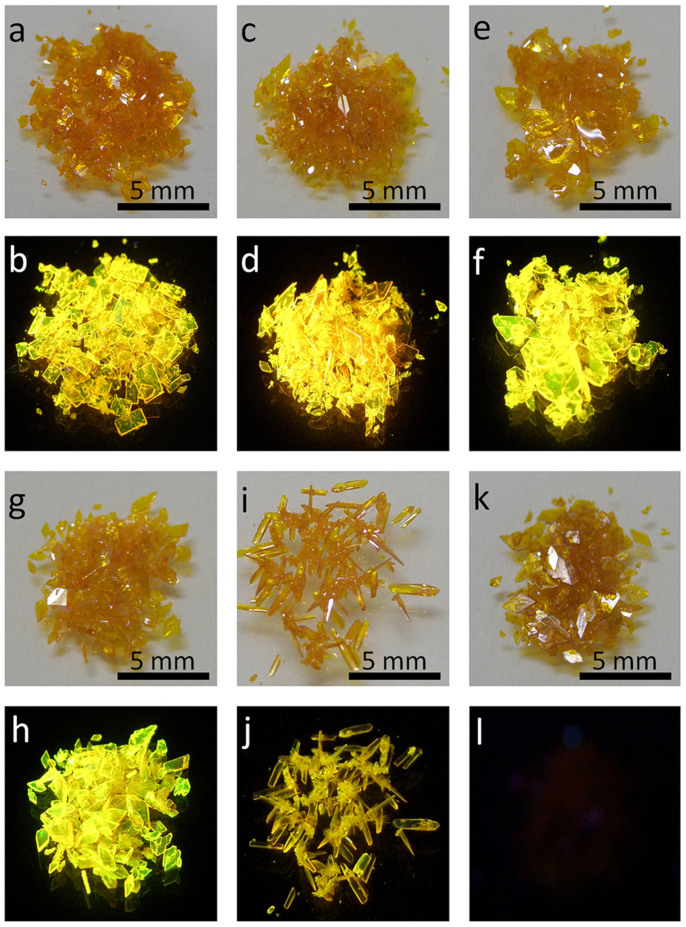
Photographic images of large amount of crystals under daylight (a, c, e, g, I, k for 1–6) and UV irradiation (b, d, f, h, j, l for 1–6).

Although **1** and **5** are known molecules, their optical properties have never been documented to data, probably due to that they are not efficient emitters in solution. Compounds **1**–**6** are poorly fluorescent in dilute CH_2_Cl_2_ (Figure 2), neat amorphous thin film and polymethyl methacrylate (PMMA) film with low doping concentration. However, their crystalline samples display conformation-dependent emission properties (Figure 3 and 4). The optical properties of compounds **1**–**6** in various states are fully investigated and those in crystalline state related to the major viewpoint of the relationship between molecular conformation and fluorescence efficiency will be focused on.

The optical properties of **1**–**6** in solution are firstly discussed. The UV-vis and fluorescent spectra of **1**–**6** in CH_2_Cl_2_ (1 × 10^−5^ M) are measured (Figure 2) and the data are outlined in Table 1. Compound **1** displays a strong absorption band centered at 433 nm, corresponding to the π → π* transition. The absorption bands of compounds **2**, **3**, **4** and **5** are slightly blue shifted by 2–5 nm and that of **6** peaking at 438 nm is red shifted by 5 nm compared to the absorption peak of **1**. The similar absorption bands in solution indicate that introducing methyl, fluoro or methoxyl groups at different positions has negligible effect on their energy gaps. These compounds are very weak green to yellow fluorescent or nearly nonluminous in solution and their solution quantum yields are quite low (0–6.3%, Table 1). The emission bands which peak in the range of 488–494 nm in CH_2_Cl_2_ are very similar, consistent with the observed trend of the absorption bands.

As mentioned above, these compounds readily form bulky crystals with very similar morphology and size. The crystals of compound **1**–**6** are yellow and have similar absorption bands as shown in Supplementary Fig. S2. However, unlike the solution samples, the crystals of these compounds display distinct different emission behavior. Crystals of compounds **1**–**4** are brightly yellow or orange fluorescent at 585, 599, 571, and 550 nm, respectively, as shown in Figure 3. The fluorescence in crystalline samples **1**–**4** is greatly red shifted compared to that of the corresponding solution samples. This observation reflects the great impact of aggregation on not only emission color but also fluorescence efficiency of these materials. The quantum yields of crystalline samples **1**–**4** are 0.39, 0.43, 0.53, and 0.42, respectively, indicating that the emissions of compounds **1**–**4** are significantly enhanced after crystallization. The crystal of **5** is moderately fluorescent at 571 nm with quantum yield of 0.17. The quantum yield of crystal **5** is greatly larger than that of its solution sample but lower than those of crystals **1**–**4**. Surprisingly, the crystal of compound **6** is nearly non-luminous (*φ*_f_ < 0.001), although the molecular structure, crystal size, shape and color are very similar between the crystals of **6** and **1**–**5**.

The fluorescence of these compounds in spin-coated films in which molecules amorphously aggregate is also measured. The films are green to yellow emissive (529–588 nm) with quite low emission intensity (Supplementary Fig. S3a). Therefore, the emission enhancements of **1**–**5** only take place when molecules regularly aggregate. Thin film of PMMA with 2 wt% **1** is nearly non-luminous (Supplementary Fig. S3b), demonstrating the strong emission of crystals is due to not only restriction of intramolecular rotation (RIR) but also some other factors. Therefore, the emission behaviors of **1**–**6** in crystal forms are very interesting and worth deep investigating.

### Crystal structures

**Figure 5:**
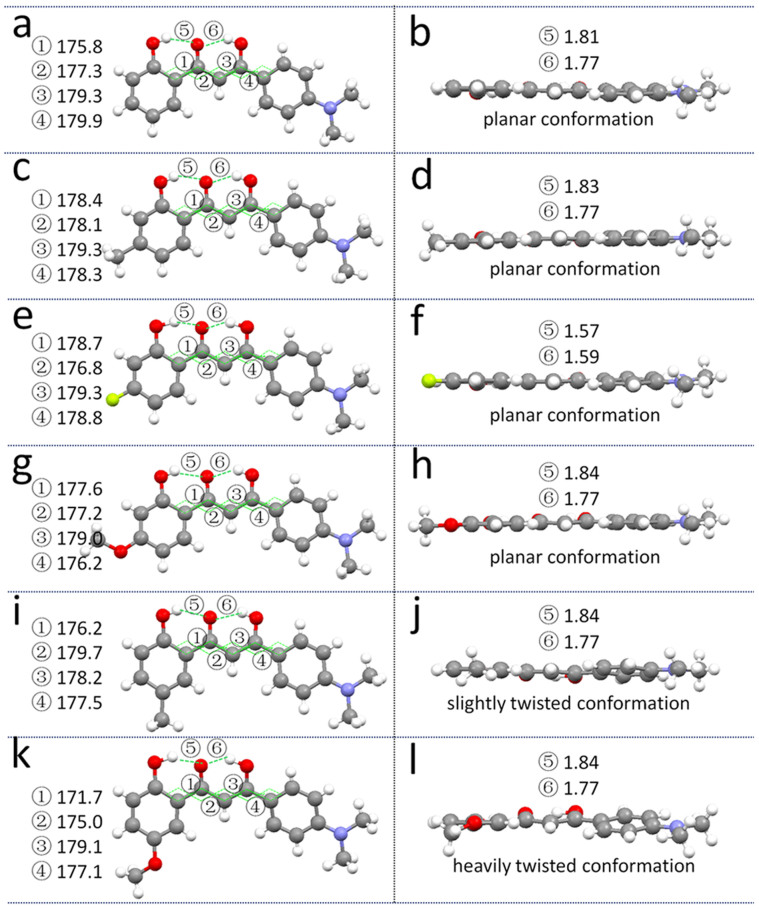
Molecular conformations of the individual molecules in crystals 1–6 (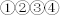: torsion angle/° and 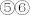: hydrogen bond length/Å).

**Figure 6:**
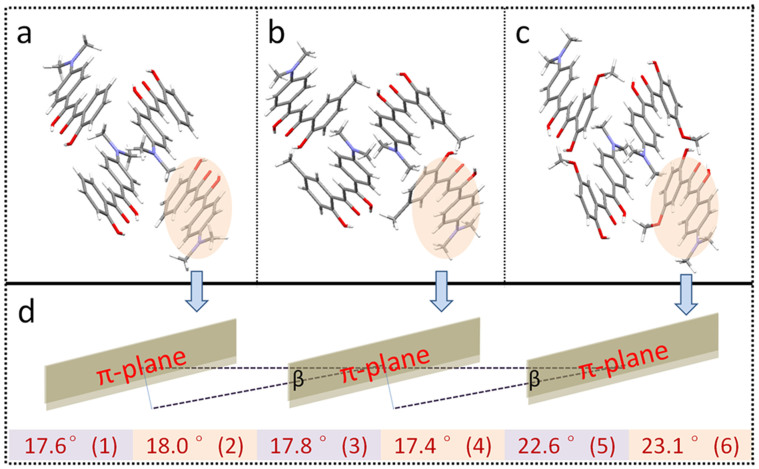
Molecular packing structures of crystals 1 (a), 5 (b), 6 (c) along the crystallographic a direction (Highlighted: J-type stacked chains) and schematic diagram of J-aggregated chains together with pitch angles of 1–6 (d).

For a deep understanding of the different emission intensities, crystal structures of **1**–**6** are carefully investigated and compared (CCDC (Cambridge Crystallographic Data Centre) numbers: 1020916-1020921). Crystals **1**–**6** hold the same crystal system (monoclinic) as well as space group (P2_1_/c) and contain one individual molecule in the unit cell. The molecular conformations with torsion angles between out benzene rings and crystal packing structures along crystallographic *a* direction are shown in Figure 5 and 6, respectively. As shown in Fig. 5b, each individual molecule in crystal **1** can form very strong intramolecular hydrogen bonds between the hydroxyl and carbonyl groups (O–H**···**O distances: 1.81 Å and 1.77 Å). The hydrogen bonds are beneficial to the molecular rigidity which effectively facilitates the molecular planarization. The constituent molecule in crystal **1** takes a rather planar conformation with four torsion angles (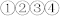) close to 180°. Additionally, each molecule connects to four adjacent molecules through C–H**···**π interactions and intermolecular hydrogen bonds with distances of 2.73 Å and 2.71 Å, respectively (Supplementary Fig. S4a). In the crystal packing structure, molecules pack into a typical J-aggregation mode which further forms a cross-shape network (Supplementary Fig. S4b). This type of molecular arrangement successfully avoids π–π overlapping that would inevitably have adverse effect for the fluorescence efficiency. The rigid planar structure together with the J-type packing makes the crystal highly fluorescent.

Crystals **2**–**4** with methyl, fluoro and methoxyl group at Para position relative to the carbonyl group take similar planar conformations as crystals **1** (Figure 5). These comparisons indicate that introducing substituent at the Para position has negligible effect on the molecular conformation of the formed crystal samples. The crystals of **5** with methyl and **6** with methoxyl groups at the Meta position display different molecular conformations compared with crystals **1**–**4**. The constituent molecule of crystal **5** shows a slightly distorted conformation. Interestingly, more distorted molecular structure is detected due to the collaborative action of molecular rotations around four carbon-carbon bonds between two benzene rings in crystal **6**. However, all the crystals take very similar molecular packing structures (Supplementary Fig. S5). The comparison of packing structures among **1**, **5**, and **6** along crystallographic *a* direction is outlined in Figure 6a–c. The pitch angles of neighboring molecules in the J-type stacked structures are very small and comparable (Figure 6d). The conformation feature of molecules **1**–**6** can be classified into three different fashions, **1**–**4** take rather planar skeleton, **5** holds a slightly bent structure and **6** possesses a heavily twisted conformation. These differences do not induce different molecular packing structures but dramatically affect emission efficiencies of the crystal samples.

### Molecular conformation-quantum yield relationship

According to the crystallographic analysis, the packing modes and molecular orientations of crystals **1**–**6** are identical. The substituents like methyl, fluoro, and methoxyl do not influence the optical properties of the individual molecule in solution on the basis of the emission and absorption measurement. The crystal size and shape, which can affect the fluorescence efficiency in a certain degree, are comparable among these crystals. As a consequence, all the possible factors that may impact crystal quantum yields can be ruled out except molecular conformation. In this sense, the huge difference of quantum yields is assigned to the different molecular conformations.

**Figure 7:**
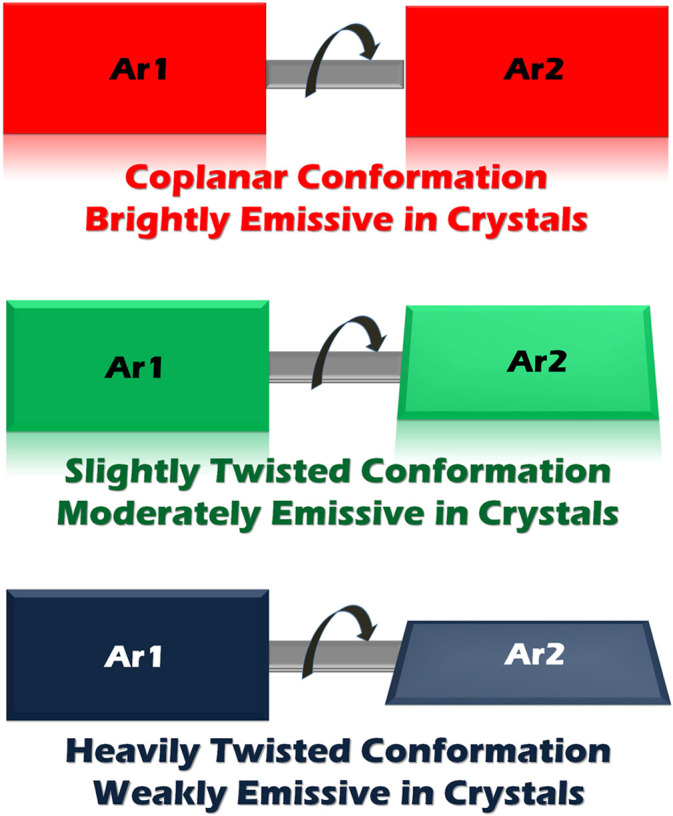
Schematic diagram of molecular conformation-fluorescence efficiency relationship.

The individual molecules of the highly fluorescent crystals **1**–**4** take a rather planar molecular conformation as a joint action of four torsion angles between two benzene rings at both sides of the molecules. However, crystal **5** contains the molecule with slightly twisted conformation. Significantly, the molecules in crystal **6** obviously deviate from the planar skeleton and display relatively large torsion angles. Crystals **1**–**4** holding planar molecular conformation show high fluorescence efficiency, crystal **5** with slightly twisted structure exhibits moderate emission and crystal **6** with heavily bent conformation is almost not emissive. Thus, the fluorescence of crystalline samples **1**–**6** is uniquely related to the conformation of the constituent molecule (Figure 7). The more planar conformation the individual molecule adopts, the brighter emission the crystal sample has. The role that induces conformation variation of this kind of molecule can also be drawn out on the basis of the molecular conformation-quantum yield relationship. Crystals **1**–**4** have very similar molecular conformations, implying that substituent at Para position can only finely tune the torsion angles and thus is not the key factor in determining the change of the efficiency. Crystals **4** and **6** with the same methoxyl groups locating at different positions give rise to completely different molecular conformations, reflecting substitution positions on the backbone play a dominate role on molecular planarity, thus the fluorescence efficiency. Different groups such as methyl (**5**) and methoxyl (**6**) locating at Meta position show different effects compared to those substituted at Para position, suggesting that the influence of the functionalization group on conformation is somewhat dependent on substitution positions.

### Mechanism considerations on emission behaviors

**Figure 8:**
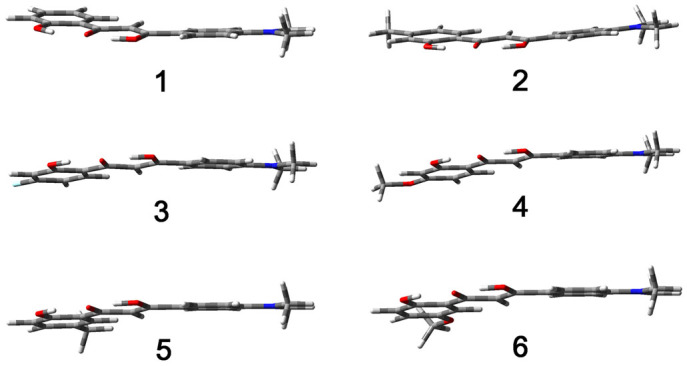
Optimized ground-state molecular structures of compounds 1–6.

The present molecules with slightly changed substituents produce crystals with similar morphologies but significantly different fluorescence efficiencies. The main factor that affects the emission intensities of these crystals is considered to be the molecular conformation. To demonstrate this point, theoretical calculations on the ground state in the gas phase have been carried out using the CAM-B3LYP functional with the 6-31G(d,p) basis sets. For compounds **1**–**6**, the HOMO and LUMO are both delocalized on the entire molecule, indicating effective conjugation of the π-electrons in the ground state (Supplementary Fig. S6). Substituted groups have certain effect on HOMO and LUMO energy levels, as shown in Table S1. However, the energy gaps are very similar among compounds **1**–**6**. These results are perfectly consistent to the observed similar optical properties of these compounds in solution. Molecules of **1**–**6** take a slightly twisted and bent structure in the ground state based on the calculation results (Figure 8). The twisted molecular structure character is not beneficial for luminescent materials. Correspondingly, all these compounds are indeed poorly emissive in solutions. In crystalline forms, the individual molecules of **1**–**4** hold rather planar skeletons which are obviously different to the calculated ground-state molecule structures. Thus, conformation planarization occurs when the molecules of **1**–**4** aggregate from solution into crystalline forms. However, the molecular conformations of compounds **5** and **6** slightly and heavily derivate from planar form, respectively, revealing that the occurrence of molecular planarization is affected by substituted groups. All the molecules form very similar infinite J-type chain structure, despite the difference of conformations. The J-type packed molecule chains further form cross-shape stacking structures through weak non-covalent interactions. The closely packing structure with both J-aggregation and cross-shape manners as well as rich intermolecular interactions may strictly confine the molecular rotations as well as effectively reduce non-radiative transition rate of the excited species. Therefore, the crystalline samples **1**–**4** with planar constituent molecules exhibit bright luminescence. Molecules **5** and **6** cannot take the flat skeleton in crystals and thus fluorescence efficiencies of crystalline samples **5** and **6** significantly reduce.

### Amplified spontaneous emissions of crystals 1–5

Amplified spontaneous emission (ASE) due to waveguiding in single crystals has recently attracted great attention since this phenomenon was firstly disclosed in α-octithiophene crystals by Nunzi in 1997^44^. High quality single crystals accompanied with co-planar facets and high fluorescence efficiencies are possible candidates for generating ASE. Although many brightly fluorescent organic crystals have been synthesized by structural modification and crystal engineering, single crystals showing low threshold values as well as significantly narrowed emissions are so far very rare^45^,^46^,^47^. Interestingly, the fluorescent crystals **1**–**5** presented in this work show stronger emission in the edge area than their bodies as shown in Figure 3. The self-waveguided emissions in the crystals are usually considered as the prerequisite for ASE or lasing. To test this possibility, a slice of crystal **1** with size of about 2 mm × 4 mm × 10 μm was excited with a pulsed lasing beam and the emission spectra were collected in the edge area.

**Figure 9:**
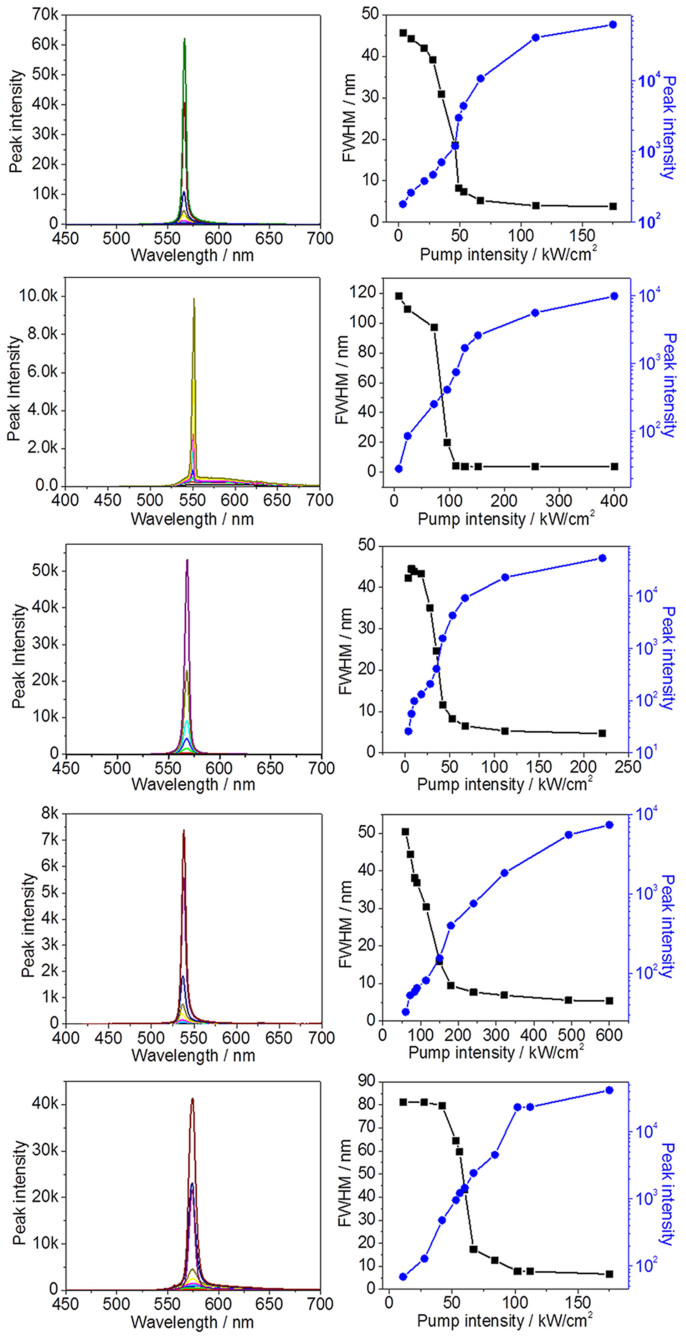
PL spectra of crystals 1–5 as a function of the pump laser energy and dependence of the peak intensity and FWHM of emission spectra on the pump laser energy.

Figure 9 shows the dependence of the peak intensity and the full widths at half-maximum (FWHM) on the pump energy. When the pump energy is less than a certain degree, the emission spectra are featured as a broad band with a FWHM over 45 nm. As the pump energy increases, the emission intensity displays a sharp increase accompanied with an obviously narrowed emission band with the FWHMs from 45 nm to 3.8 nm. The nonlinear curves characterized by the peak intensity and pump energy demonstrate the ASE behavior of the crystal. The threshold value is about 39 kW/cm^2^ calculated by the slope change of FWHM/peak intensity versus pump energy curve. The other fluorescent crystals **2**–**5** which hold similar molecular packing structures also display ASE character. Supplementary Fig. S7 shows the fluorescent microphotograph of the crystals, indicating the quality and surface neatness of crystals of compounds **2** and **4** are poor compared with those of the other three crystals of compounds **1**, **3** and **5**. There are many cracks in crystals of compounds **2** and **4** which make the light scatter out from the crystal body. As a result, crystals of compounds **2** and **4** have larger threshold values compared with those of compounds **1**, **3** and **5**. The threshold values for compounds **1**–**5** are 39, 71, 38, 109 and 46 kW/cm^2^, respectively, which are comparable to those of the typical reported organic crystals (Supplementary Table S2). The polarized emission from the crystal edge area of compound **1** was measured at the pumping laser energy of about 200 μJ·pulse^−1^·cm^−2^ by using a polarizer in front of the optical fiber. Figure S8a shows the dependence of the emission intensity on the relative porlarization angle φ. The difference between the angles corresponding to the maximum and the minimum of emission intensity in the fitting curve is approximately 90 degree, in coincidence with the highly ordered packing alignment of molecules in the crystal. The gain coefficient of compound **1** was measured by adjusting the pump stripe length with a slit. As shown in Figure S8b, the emission band becomes narrower with exponentially grown peak intensity as increasing the pump stripe length. Increasing the pump energy can accelerate the emission band narrowing which is in consistent with the ASE theory. The maximum of the gain coefficient is about 55 cm^−1^ under 567 kW/cm^2^ (2835 μJ·pulse^−1^·cm^−2^) pump intensity (Supplementary Fig. S8c). Notably, all these crystals display very small FWHM in the range of 3.64–6.59 nm (Table 2), strongly indicating their potentials as organic crystal lasing media.

In summary, we here report a model that precisely discloses the molecular conformation-fluorescence efficiency relationship of organic materials on the basis of careful analysis on crystal structures as well as emission properties. Previously reports about the influence of molecular conformation on emission properties usually accompanied with contributions from molecular packing effect. Our observations in the present system indicate the quite different fluorescence efficiencies among crystals **1**, **5**, and **6** are merely originated from the change of molecular conformations since all the crystals hold the same crystal system, space group, packing structure as well as similar crystal morphology. In addition, the fluorescent crystals **1**–**5** show perfect ASE behaviors as a result of suitable crystal morphology as well as ideal molecular arrangement. We believe these results might not only have significant impact on the key topic of structure-property relationship in material science but also provide guidance for constructing organic lasing crystals.

## Methods

### Materials

All chemicals were obtained from Acros, Sigma-Aldrich or TCI Chemical Co. at the highest purity available. Absolute solvents (over molecular sieves) and starting materials obtained from commercial source were used without further purification.

### Instrumentation

NMR spectra were determined on a Bruker Avance 500 MHz spectrometer with tetramethylsilane as the internal standard. Mass spectra were recorded on a GC/MS mass spectrometer. UV−vis absorption spectra were recorded by a Shimadzu UV-2550 spectrophotometer with (for crystals) or without (for solution) an integrating sphere. The emission spectra were recorded using a Shimadzu RF-5301 PC spectrometer or a Maya2000 Pro CCD spectrometer. The absolute fluorescence quantum yields were measured on Edinburgh FLS920 using an integrating sphere. For the laser test, the crystal slices were irradiated by the third harmonic (355 nm) of a Nd:YAG (yttrium–aluminum–garnet) laser at a repetition rate of 10 Hz and pulse duration of about 5 ns. The energy of the pumping laser was adjusted by using the calibrated neutral density filters. The beam was focused into a stripe whose shape was adjusted to 2 × 0.5 mm by using a cylindrical lens and a slit. The edge emission and PL spectra of the crystals were detected using a Maya2000 Pro CCD spectrometer. The polarization of light emitted from the edge of the crystal was measured by rotating a polarizer. All the measurements were carried out at room temperature under ambient conditions.

### Single crystal structure

Single crystal X-ray diffraction data were collected on a Rigaku RAXIS-PRID diffractometer using the ω-scan mode with graphite-monochromator Mo Kα radiation. The structures were solved with direct methods using the SHELXTL programs and refined with full-matrix leastsquares on *F*^2^. Non-hydrogen atoms were refined anisotropically. The positions of hydrogen atoms were calculated and refined isotropically.

## Author Contributions

H.Z. and Y.W. conceived and designed the experiments. X.C., S.H., Y.Z., F.L., K.Y., C.J. and J.W. performed all experiments. H.Z. and X.C. were mainly responsible for preparing the manuscript. All the authors discussed the results and reviewed the manuscript.

## Supplementary Material

Supplementary Information Supplementary Information

## Figures and Tables

**Figure 1:**
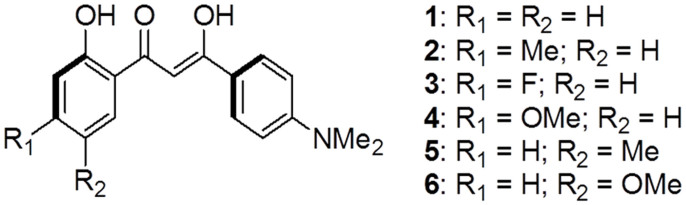
Molecular structures of compounds 1–6.

**Table 1:** Optical data of 1–6 in different phases

compound	*λ*_abs_/nma)	*λ*_em_/nm (*φ_f_*)b)	*λ*_em_/nm (*φ_f_*, τ/ns)c)
1	433	494(0.94%)	585(39%, 2.12)
2	430	489(1.01%)	599(43%, 2.39)
3	430	493(6.32%)	571(53%, 2.66)
4	428	488(2.79%)	550(42%, 1.80)
5	431	488(1.34%)	571(17%, 1.05)
6	437	490(<0.1%)	d)

^a)^in CH_2_Cl_2_ solution (1 × 10^−5^ м); ^b)^ in CH_2_Cl_2_ solution (1 × 10^−5^ м); ^c)^ in crystalline state; ^d)^ not determined.

**Table 2:** Threshold values and minimum FWHMs of crystals 1–5

compound	1	2	3	4	5
Threshold (μJ·cm^−2^·pulse^−1^)	195	355	190	545	230
Minimum FWHM (nm)	3.85	3.64	4.71	5.32	6.59

## References

[b1] Reineke S. *et al.* White organic light-emitting diodes with fluorescent tube efficiency. Nature 459, 234–238 (2009). 19444212 10.1038/nature08003

[b2] Lee S. H., Jang B.-B. & Kafafi Z. H. Highly fluorescent solid-state asymmetric spirosilabifluorene derivatives. J. Am. Chem. Soc. 127, 9071–9078 (2005). 15969585 10.1021/ja042762q

[b3] Xie Z. *et al.* Cross dipole stacking in the crystal of distyrylbenzene derivative: the approach toward high solid-state luminescence efficiency. J. Am. Chem. Soc. 127, 14152–14153 (2005). 16218587 10.1021/ja054661d

[b4] Kim Y., Bouffard J., Kooi S. E. & Swager T. M. Highly emissive conjugated polymer excimers. J. Am. Chem. Soc. 127, 13726–13731 (2005). 16190739 10.1021/ja053893+

[b5] Friend R. H. *et al.* Electroluminescence in conjugated polymers. Nature 397, 121–128 (1999).

[b6] Zhao C.-H., Wakamiya A., Inukai Y. & Yamaguchi S. Highly emissive organic solids containing 2,5-diboryl-1,4-phenylene unit. J. Am. Chem. Soc. 128, 15934–15935 (2006). 17165696 10.1021/ja0637550

[b7] Wakamiya A., Mori K. & Yamaguchi S. 3-Boryl-2,2′-bithiophene as a versatile core skeleton for full-color highly emissive organic solids. Angew. Chem. Int. Ed. 46, 4273–4276 (2007). 10.1002/anie.200604935 17378005

[b8] Zhang C. *et al.* Two-photon pumped lasing in single-crystal organic nanowire exciton polariton resonators. J. Am. Chem. Soc. 133, 7276–7279 (2011). 21517020 10.1021/ja200549v

[b9] Mizuno H., Ohnishi I., Yanagi H., Sasaki F. & Hotta S. Lasing from epitaxially oriented needle crystals of a thiophene/phenylene co-oligomer. Adv. Mater. 24, 2404–2408 (2012). 22492301 10.1002/adma.201104182

[b10] Wang H. *et al.* Cyano-Substituted Oligo(p-phenylene vinylene) Single Crystals: A Promising Laser Material. Adv. Funct. Mater. 21, 3770–3777 (2011).

[b11] Li X., Gao N., Xu Y., Li F. & Ma Y. Self-cavity laser oscillations with very low threshold from a symmetric organic crystal waveguide. Appl. Phys. Lett. 101, 063301 (2012).

[b12] Yanagi H., Ohara T. & Morikawa T. Self-waveguided gain-narrowing of blue light emission from epitaxially oriented *p*-sexiphenyl crystals. Adv. Mater. 13, 1452–1455 (2001).

[b13] Rabbani-Haghighi H., Forget S., Chénais S. & Siove A. Highly efficient, diffraction-limited laser emission from a vertical external-cavity surface-emitting organic laser. Opt. Lett. 35, 1968–1970 (2010). 20548355 10.1364/OL.35.001968

[b14] Wang H. *et al.* Doped organic crystals with high efficiency, color-tunable emission toward laser application. Crystal Growth & Design 9, 4945–4950 (2009).

[b15] Lin J.-Y. *et al.* A rational molecular design of β-phase polydiarylfluorenes: synthesis, morphology, and organic Lasers. Macromolecules 47, 1001–1007 (2014).

[b16] Wei Y. & Chen C. T. Doubly ortho-linked cis-4,4′-bis(diarylamino)stilbene/fluorene hybrids as efficient nondoped, sky-blue fluorescent materials for optoelectronic applications. J. Am. Chem. Soc. 129, 7478–7479 (2007). 17523643 10.1021/ja070822x

[b17] Liao S.-H. *et al.* Hydroxynaphthyridine-derived group III metal chelates: wide band gap and deep blue analogues of green Alq_3_ (tris(8-hydroxyquinolate)aluminum) and their versatile applications for organic light-emitting diodes. J. Am. Chem. Soc. 131, 763–777 (2009). 19093863 10.1021/ja807284e

[b18] Shimizu M. *et al.* 1,4-Bis(diarylamino)-2,5-bis(4-cyanophenylethenyl)benzenes: fluorophores exhibiting efficient red and near-infrared emissions in solid state. Angew. Chem. Int. Ed. 51, 4095–4099 (2012). 10.1002/anie.201108943 22422698

[b19] Wang K. *et al.* High-performance red, green, and blue electroluminescent devices based on blue emitters with small singlet–triplet splitting and ambipolar transport property. Adv. Funct. Mater. 23, 2672–2680 (2013).

[b20] Liu L. *et al.* Efficient solution-processed blue phosphorescent organic light-emitting diodes with halogen-free solvent to optimize the emissive layer morphology. Org. Electron. 15, 1401–1406 (2014).

[b21] Tsiminis G. *et al.* Nanoimprinted organic semiconductor laser pumped by a light-emitting diode. Adv. Mater. 25, 2826–2830 (2013). 23580437 10.1002/adma.201205096

[b22] Yu L. Polymorphism in molecular solids: an extraordinary system of red, orange, and yellow crystals. Acc. Chem. Res. 43, 1257–1266 (2010). 20560545 10.1021/ar100040r

[b23] Wuest J. D. Molecular solids: co-crystals give light a tune-up. Nat. Chem. 4, 74–75 (2012). 22270638 10.1038/nchem.1256

[b24] Fan G. & Yan D. Positional isomers of cyanostilbene: two-component molecular assembly and multiple-stimuli responsive luminescence. Sci. Rep. 4, 4933; 10.1038/srep04933 (2014). 24816686 PMC5381361

[b25] Mutai T., Tomoda H., Ohkawa T., Yabe Y. & Araki K. Switching of polymorph-dependent ESIPT luminescence of an imidazo[1,2-a]pyridine derivative. Angew. Chem. Int. Ed. 47, 9522–9524 (2008). 10.1002/anie.200803975 18972481

[b26] Malwitz M. A. *et al.* Crystallization and interconversions of vapor-sensitive, luminescent polymorphs of [(C_6_H_11_NC)_2_Au^I^](AsF_6_) and [(C_6_H_11_NC)_2_Au^I^](PF_6_). J. Am. Chem. Soc. 2012, *134*, 10885–10893 (2012). 22506844 10.1021/ja302025m

[b27] Abe Y., Karasawa S. & Koga N. Crystal structures and emitting properties of trifluoromethylaminoquinoline derivatives: thermal single-crystal-to-single-crystal transformation of polymorphic crystals that emit different colors. Chem. Eur. J. 18, 15038–15048 (2012). 23032710 10.1002/chem.201201213

[b28] Judith Percino M. *et al.* Important role of molecular packing and intermolecular interactions in two polymorphs of (*Z*)-2-phenyl-3-(4-(pyridin-2-yl)phenyl)acrylonitrile. preparation, structures, and optical properties. J. Mol. Struct. 10.1016/j.molstruc.2014.04.088 (2014).

[b29] Varughese S. Non-covalent routes to tune the optical properties of molecular materials. J. Mater. Chem. C 2, 3499–3516 (2014).

[b30] Harada N., Abe Y., Karasawa S. & Koga N. Polymorphic equilibrium responsive thermal and mechanical stimuli in light-emitting crystals of N-methylaminonaphthyridine. Org. Lett. 14, 6282–6285 (2012). 23234438 10.1021/ol302963e

[b31] Zhang H., Zhang Z., Ye K., Zhang J. & Wang Y. Organic crystals with tunable emission colors based on a single organic molecule and different molecular packing structures. Adv. Mater. 18, 2369–2372 (2006).

[b32] Li Y. *et al.* Extended π-conjugated molecules derived from naphthalene diimides toward organic emissive and semiconducting materials. J. Org. Chem. 78, 2926–2934 (2013). 23461275 10.1021/jo302677k

[b33] Wang C. *et al.* Polymorph, assembly, luminescence and semiconductor properties of a quinacridone derivative with extended π-conjugated framework. J. Mater. Chem. C 1, 5548–5556 (2013).

[b34] Vanormelingen W., Pandey L., Auweraer M. V., Verbiest T. & Koeckelberghs G. Steering the conformation and chiroptical properties of poly(dithienopyrrole)s substituted with chiral OPV side chains. Macromolecules 43, 2157–2168 (2010).

[b35] Qiu J.-X. *et al.* Substitution degree engineering the crystal packing and optoelectronic properties of benzofuranvinyl-substituted benzene-cored derivatives. J. Mater. Chem. C 2, 5954–5962 (2014).

[b36] Schwoerer M. & Wolf H. C. [Electroluminescence and the Photovoltaic Effect.] Organic Molecular Solids [Schwoerer, M. & Wolf, H. C.] [365–390] (Wiley-VCH Verlag GmbH, Weinheim, Germany, 2007).

[b37] Mutai T., Satou H. & Araki K. Reproducible on–off switching of solid-state luminescence by controlling molecular packing through heat-mode interconversion. Nat. Mater. 4, 685–687 (2005). 16113683 10.1038/nmat1454

[b38] Zhang G., Lu J., Sabat M. & Fraser C. L. Polymorphism and reversible mechanochromic luminescence for solid-state difluoroboron avobenzone. J. Am. Chem. Soc. 132, 2160–2162 (2010). 20108897 10.1021/ja9097719

[b39] Wang Y. *et al.* Naphthyl-Fused π-Elongated Porphyrins for Dye-Sensitized TiO_2_ Cells. J. Phys. Chem. C 116, 15576–15585 (2012).

[b40] Zhao Y. *et al.* Thermally induced reversible phase transformations accompanied by emission switching between different colors of two aromatic-amine compounds. Adv. Mater. 21, 3165–3169 (2009).

[b41] Wang K. *et al.* Organic polymorphs: one-compound-based crystals with molecular-conformation- and packing-dependent luminescent properties. Adv. Mater. 26, 6168–6173 (2014). 25069771 10.1002/adma.201401114

[b42] Kedar R. M., Vidhale N. N. & Chincholkar M. M. Synthesis of 3,4,5-trisubstituted pyrazolines and their antimicrobial study. Oriental Journal of Chemistry 13, 249–252 (1997).

[b43] Kormann C. *et al.* Diarylpropane-1,3-dione derivatives as tetR-inducing tetracycline mimetics: synthesis and biological investigations. ChemBioChem 10, 2924–2933 (2009). 19885899 10.1002/cbic.200900564

[b44] Fichou D., Delysse S. & Nunzi J.-M. First evidence of stimulated emission from a monolithic organic single crystal: α-Octithiophene. Adv. Mater. 9, 1178–1181 (1997).

[b45] Ichikawa M. *et al.* Improved crystal-growth and emission gain-narrowing of thiophene/phenylene co-oligomers. Adv. Mater. 15, 213–217 (2003).

[b46] Ichikawa M. *et al.* Photopumped laser oscillation and charge-injected luminescence from organic semiconductor single crystals of a thiophene/phenylene co-oligomer. Appl. Phys. Lett. 87, 221113 (2005).

[b47] Qu S., Lu Q., Wu S., Wang L. & Liu X. Two dimensional directed π-π interactions in a linear shaped bi-1,3,4-oxadiazole derivative to achieve organic single crystal with highly polarized fluorescence and amplified spontaneous emissions. J. Mater. Chem. 22, 24605–24609 (2012).

