# Supplementary material for: Emission behaviors of unsymmetrical 1,3-diaryl-β-diketones: A model perfectly disclosing the effect of molecular conformation on luminescence of organic solids
Source: Sci Rep. 2015 Mar 16;5:9140. doi: 10.1038/srep09140 (PMC4360484; doi:10.1038/srep09140)
Supplement: Supplementary Information [file srep09140-s1.pdf]

Supplementary Information for:

Emission behaviors of unsymmetrical 1,3-diaryl-β-diketones: A model perfectly disclosing the effect of molecular conformation on luminescence of organic solids

*Xiao Cheng, Feng Li, Shenghua Han, Yufei Zhang, Chuanjun Jiao, Jinbei Wei, Kaiqi Ye, Yue Wang and Hongyu Zhang**

State Key Laboratory of Supramolecular Structure and Materials, College of Chemistry, Jilin University, Qianjin Avenue 2699, Changchun 130012, P. R. China

E-mail: hongyuzhang@jlu.edu.cn

Table of contents

Part 1. Synthetic Procedure and Spectra S2−S5

Part 2. Molecular Packing Structures S6

Part 3. Theoretical Calculations S7

Part 4. Threshold Values and Fluorescent Microphotograph S8

Part 5. Emission Polarization and Gain Coefficient S9

Part 6. Crystal Data and Structure Refinement S10−S11

Part 7. ^{1}H and ^{13}C NMR Spectra S12−S19

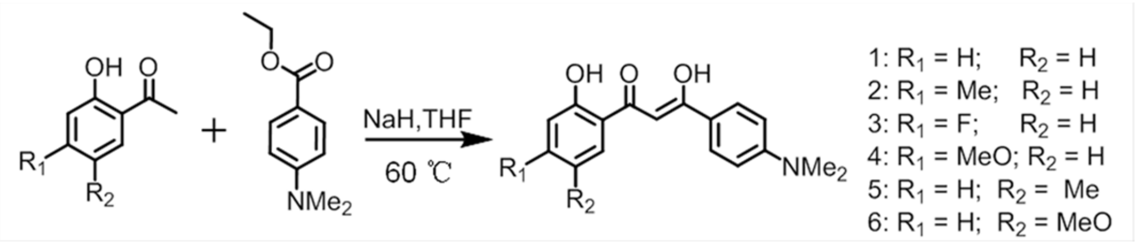

Figure S1. Synthetic procedure of complexes **1–6**.

*3-Hydroxy-3-(4-dimethylaminophenyl)-1-(2-hydroxy-4-methylphenyl)-propenone, **2***: Yield: 60%. ^{1}H NMR (500 MHz, $CDCl_3$, δ): enol tautomer (~66%) 15.94 (s, 1H), 12.29 (s, 1H), 7.92 (d, *J* = 9.5 Hz, 1H), 7.86 (d, *J* = 2.0 Hz, 2H), 6.79 (s, 1H), 6.71 (m, 3H), 6.68 (s, 1H), 3.08 (s, 6H), 2.35 (s, 3H); diketone tautomer (~34%) 12.08 (s, 1H), 7.74 (d, *J* = 8.0 Hz, 2H), 7.63 (d, *J* = 8.0 Hz, 2H), 6.77 (s, 1H), 6.71 (m, 1H), 6.66 (m, 2H), 4.50 (s, 2H), 3.07 (s, 6H), 2.33 (s, 3H). ^{13}C NMR (125 MHz, $CDCl_3$, δ): 200.25, 193.32, 190.91, 178.35, 162.92, 162.19, 153.85, 153.23, 148.51, 146.27, 131.25, 131.18, 128.71, 128.03, 120.52, 120.14, 118.73, 118.42, 116.94, 111.21, 110.76, 89.51, 50.04, 40.06, 40.01, 21.99, 21.78. MS m/z: $[M]^+$ calcd for $C_{18}H_{19}NO_3$: 297.14; found: 296.95. Anal. calcd (%) for $C_{18}H_{19}NO_3$: C, 72.71; H, 6.44; N, 4.71; found: C, 73.00; H, 6.37; N, 4.63.

*3-Hydroxy-3-(4-dimethylaminophenyl)-1-(2-hydroxy-4-fluorophenyl)-propenone, **3***: Yield: 62%. ^{1}H NMR (500 MHz, $CDCl_3$, δ): enol tautomer (~72%) 15.80 (s, 1H), 12.69 (d, *J* = 1.5 Hz, 1H), 7.18 (s, 1H), 7.88 (m, 2H), 7.74 (dd, *J* = 9.0, 6.5 Hz, 1H), 6.66 (m, 5H), 3.08 (s, 6H); diketone tautomer (~28%) 12.40 (d, *J* = 1.5 Hz, 1H), 7.88 (m, 2H), 6.66 (m, 5H), 4.49 (s, 2H), 3.08 (s, 6H). ^{13}C NMR (125 MHz, $CDCl_3$, δ): 199.81, 192.41, 190.43, 178.70, 167.69, 165.67, 164.48, 164.38, 153.94, 153.29, 134.00, 133.91, 131.29, 130.21, 130.12, 128.84, 116.20, 116.18, 111.32, 110.79,

107.00, 106.82, 105.20, 105.01, 89.41, 50.26, 40.11, 40.02. MS *m/z*: $[M]^+$ calcd for $C_{17}H_{16}FNO_3$: 301.18; found: 301.11. Anal. calcd (%) for $C_{17}H_{16}FNO_3$: C, 67.76; H, 5.35; N, 4.65; found: C, 68.00; H, 5.25; N, 4.60.

3-Hydroxy-3-(4-dimethylaminophenyl)-1-(2-hydroxy-4-methoxylphenyl)-propenone, ***4***: Yield: 64%. ^{1}H NMR (500 MHz, $CDCl_3$, δ): enol tautomer (~59%) 15.76 (s, 1H), 12.76 (s, 1H), 7.84 (d, *J* = 8.5Hz, 2H), 7.67 (d, *J* = 8.5Hz, 1H), 7.73 (dd, *J* = 7.5Hz, 2H), 6.60 (s, 1H), 6.45 (m, 2H), 3.84 (s, 3H), 3.07 (s, 6H); diketone tautomer (~41%) 12.55 (s, 1H), 7.93 (d, *J* = 9.0Hz, 2H), 7.78 (d, *J* = 9.0Hz, 1H), 6.45 (m, 1H), 6.40 (m, 1H), 3.82 (s, 3H), 3.07 (s, 6H). ^{13}C NMR (125 MHz, $CDCl_3$, δ): 198.74, 192.81, 190.98, 177.37, 166.43, 165.73, 165.22, 164.80, 153.82, 133.03, 131.30, 129.70, 128.56, 124.21, 111.42, 110.77, 107.95, 107.55, 101.32, 100.90, 89.29, 55.60, 55.49, 50.05, 40.19, 40.03. MS *m/z*: $[M]^+$ calcd for $C_{18}H_{19}FNO_4$: 313.13; found: 313.26. Anal. calcd (%) for $C_{18}H_{19}FNO_4$: C, 69.00; H, 6.11; N, 4.47; found: C, 69.39; H, 5.96; N, 4.40.

3-Hydroxy-3-(4-dimethylaminophenyl)-1-(2-hydroxy-5-methoxylphenyl)-propenone, ***6***: Yield: 52%. ^{1}H NMR (500 MHz, $CDCl_3$, δ): enol tautomer (~59%) 15.76 (s, 1H), 12.76 (s, 1H), 7.85 (d, *J* = 2.0Hz, 2H), 7.67 (d, *J* = 8.5Hz, 1H), 7.74 (dd, *J* = 9.0Hz, 2H), 6.60 (s, 1H), 6.44 (m, 2H), 3.84 (s, 3H), 3.08 (s, 6H); diketone tautomer (~41%) 12.55 (s, 1H), 7.93 (d, *J* = 9.5Hz, 2H), 7.83 (d, *J* = 2.0Hz, 1H), 6.66(d, *J* = 9.0Hz, 2H), 6.44 (m, 1H), 6.40 (d, *J* = 2.5Hz, 1H), 3.82 (s, 3H), 3.07 (s, 6H). ^{13}C NMR (125 MHz, $CDCl_3$, δ): 198.77, 192.77, 190.99, 177.41, 166.42, 165.72, 165.20, 164.79, 153.82, 153.03, 133.03, 131.28, 129.70, 128.56, 124.18, 120.37, 113.95, 112.86, 111.31,

110.75, 107.94, 107.52, 101.33, 100.91, 89.24, 55.60, 55.94, 50.00, 40.12, 40.01. MS *m/z*: $[M]^+$ calcd for $C_{18}H_{19}FNO_4$: 313.13; found: 313.31. Anal. calcd (%) for $C_{18}H_{19}FNO_4$: C, 69.00; H, 6.11; N, 4.47; found: C, 69.41; H, 5.94; N, 4.41.

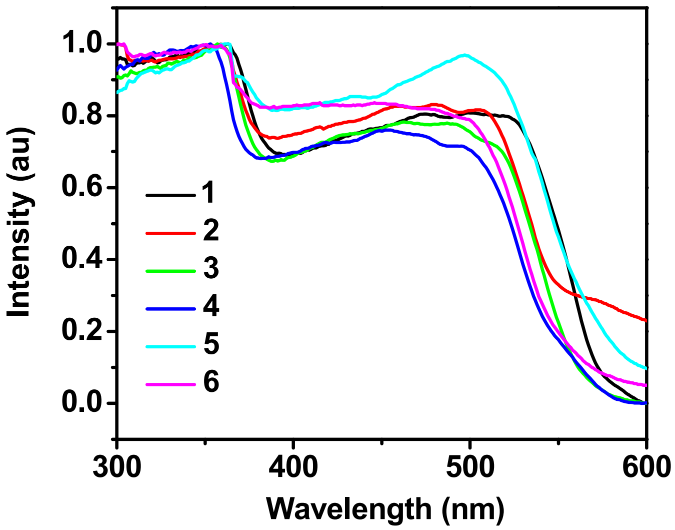

Figure S2. Absorption spectra of crystals for compounds **1–6**.

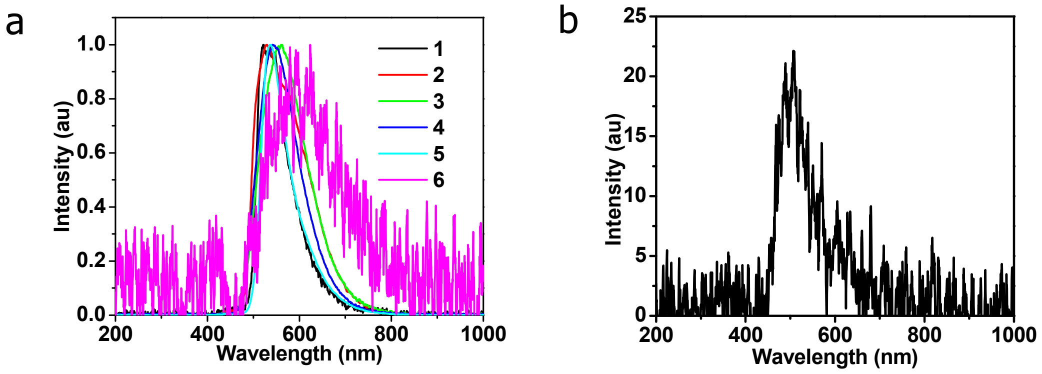

Figure S3. (a) Photoluminescent spectra of thin films for compounds **1–6**; (b) Photoluminescent spectra of thin film for compound **1** in PMMA.

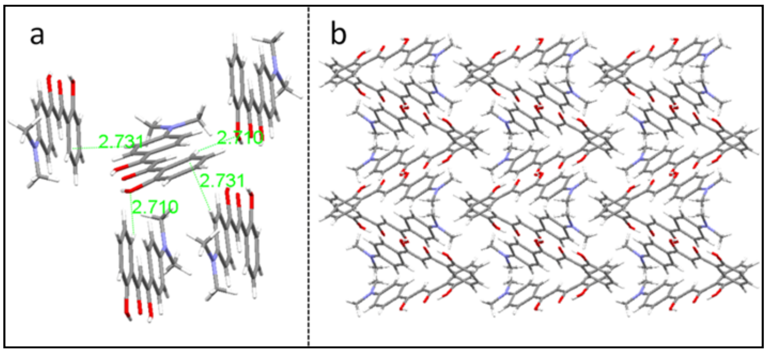

Figure S4. (a) Two types of intermolecular interaction between the adjacent molecules (/Å); (b) cross-shape network structures of crystal **1**.

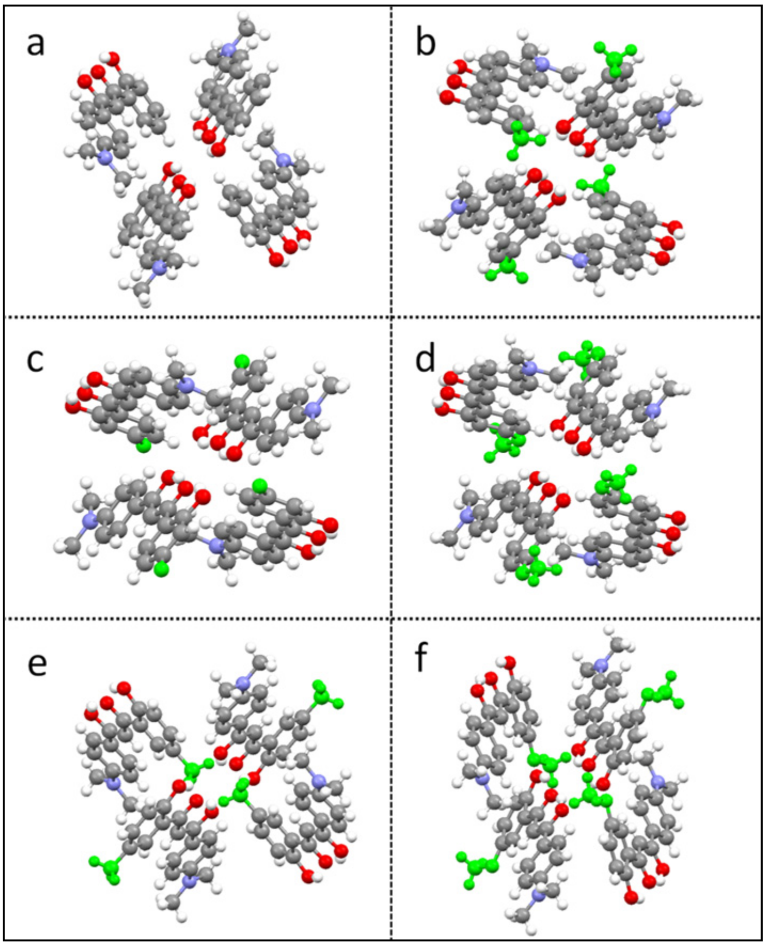

Figure S5. Molecular packing structures of crystals **1–6** (a–f) with different substituents (marked in green color).

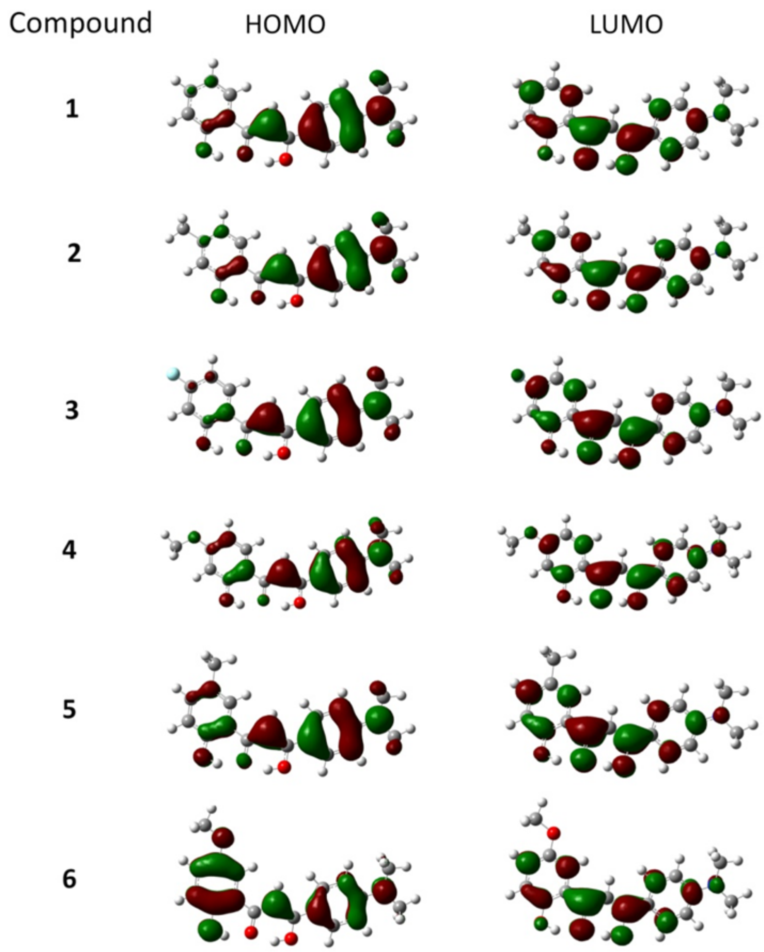

Figure S6. Theoretical calculations of HOMO, LUMO distributions on the ground state for compounds **1–6**.

Table S1. HOMO and LUMO values calculated for **1–6**.

	**1**	**2**	**3**	**4**	**5**	**6**
HOMO, eV	–6.66	–6.59	–6.70	–6.53	–6.60	–6.52
LUMO, eV	–0.65	–0.58	–0.71	–0.49	–0.62	–0.64
Eg, eV	6.01	6.01	5.99	6.04	5.98	5.98

Table S2. Threshold of ASE for different organic crystals

Compound	**1**	**2**	**3**	**4**	**5**	**AC5** (Ref 45)	**BP1T** (Ref 45)	**BP3T** (Ref 46)	**BOXD-4** (Ref 47)
Threshold value (kW/cm^2)	39	71	38	109	46	84	54	< 260	20

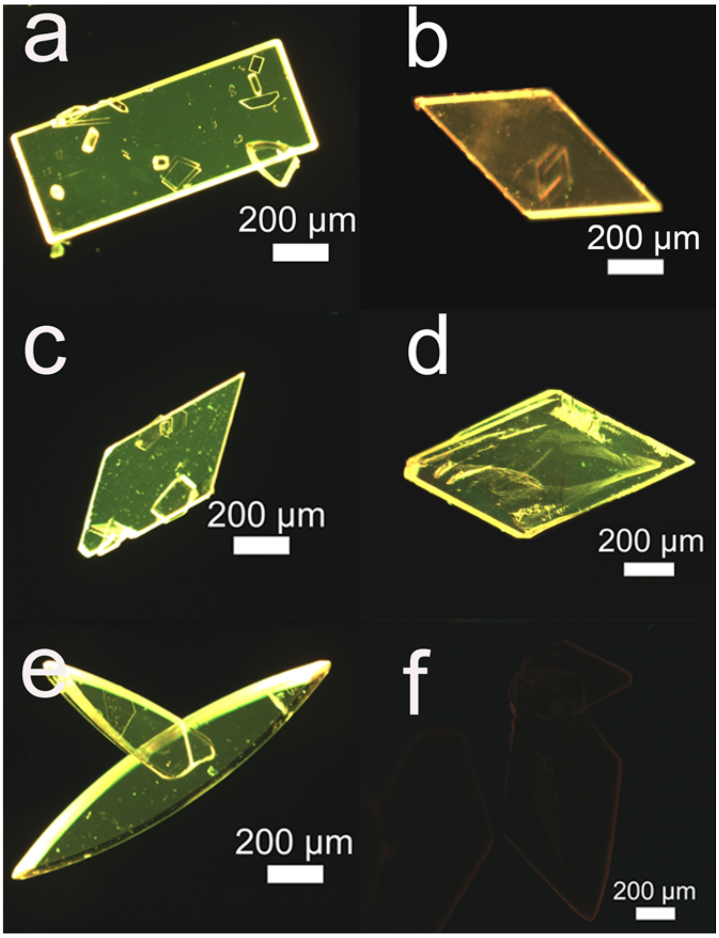

Figure S7. Fluorescent microphotograph of crystals for compounds **1–6**.

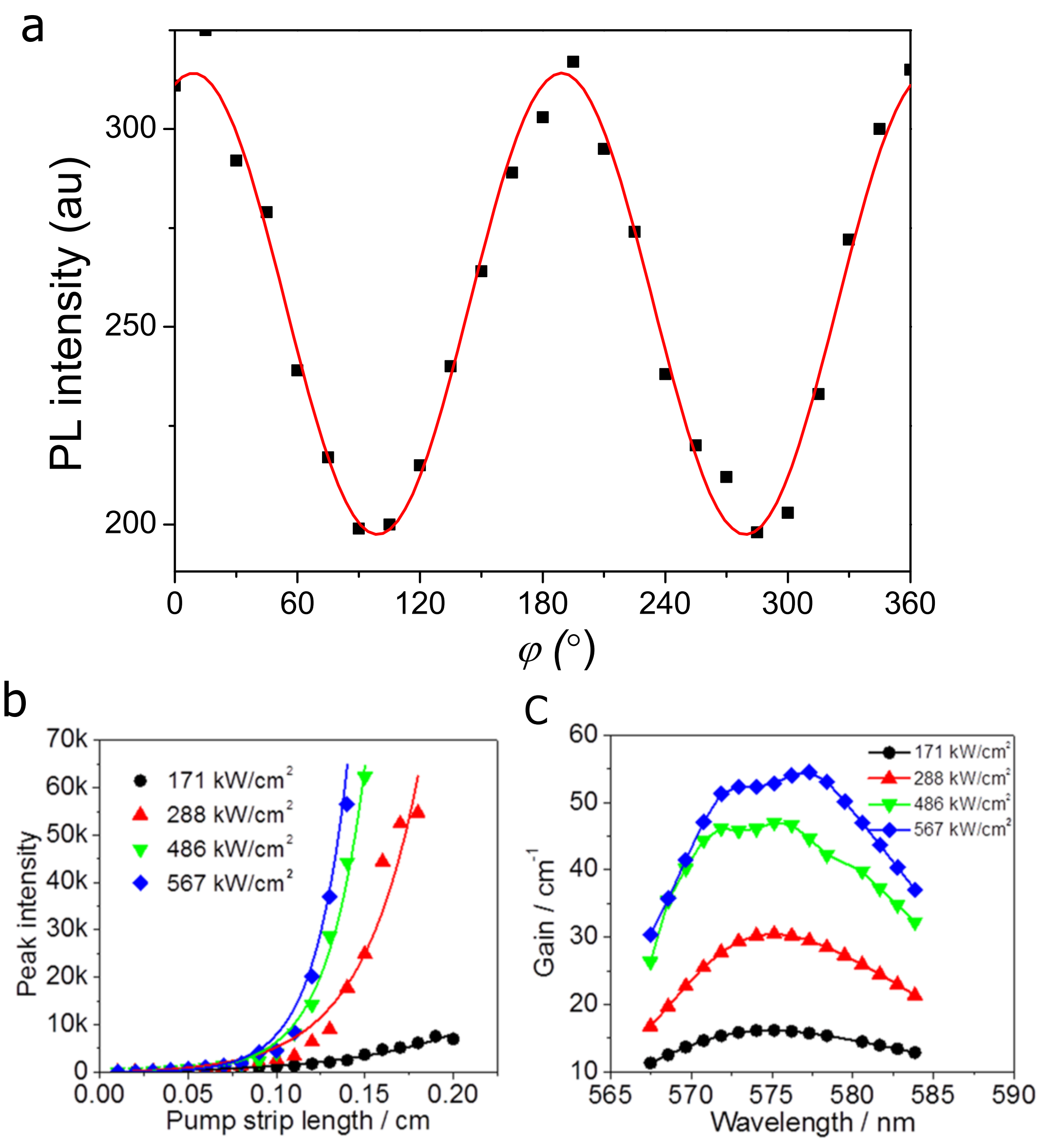

Figure S8. (a) Dependence of the intensity of porlarized light from crystal **1** on the relative porlarization angle φ at the energy of pumping laser about 200 μJ·pulse^{-1}·cm^{-2}; (b) The peak intensity of PL spectra as a function of the pump stripe length at different pump energies; (c) The net gain coefficient as a function of wavelength at different pump intensities.

Table S3. Crystal data and structure refinement for **1–6**.

Table S3-1. Crystal data and structure refinement for **1**, **2** and **3**.			
Identification code	**1**	**2**	**3**
Empirical formula	$C_{17}H_{17}NO_3$	$C_{18}H_{19}NO_3$	$C_{17}H_{16}FNO_3$
Formula weight	283.32	297.34	301.31
Temperature (K)	293(2)	293(2)	293(2)
Wavelength (Å)	0.71073	0.71073	0.71073
Crystal system, space group	Monoclinic, P2(1)/c	Monoclinic, P2(1)/n	Monoclinic, P2(1)/n
Unit cell dimensions	a = 11.877(2) Å	a = 11.795(2) Å	a = 11.957(2) Å
	b = 10.491(2) Å	b = 12.023(2) Å	b = 10.726(2) Å
	c = 12.835(3) Å	c = 12.097(2) Å	c = 12.789(3) Å
	alpha = 90 deg.	alpha = 90 deg.	alpha = 90 deg.
	beta = 117.51(3) deg.	beta = 115.79(3) deg.	beta = 117.80(3) deg.
	gamma = 90 deg.	gamma = 90 deg.	gamma = 90 deg.
Volume ($Å^3$)	1418.5(5)	1544.6(5)	1450.9(5)
Z, Calculated density (mg/m^3)	4, 1.327	4, 1.279	4, 1.379
Absorption coefficient (mm^{-1})	0.091	0.087	0.103
F(000)	600	632	632
Crystal size, mm	0.49 × 0.41 × 0.31	0.41 × 0.37 × 0.35	0.50 × 0.33 × 0.12
Theta range for data collection	3.19 to 27.48 deg.	3.21 to 27.48 deg.	3.19 to 27.48 deg.
Limiting indices	-15<=h<=14, -13<=k<=13, -15<=l<=16	-14<=h<=15, -15<=k<=15, -14<=l<=15	-15<=h<=15, -13<=k<=13, -16<=l<=16
Reflections collected / unique	13518 / 3249 [*R*(int) = 0.0370]	14815 / 3526 [*R*(int) = 0.0396]	13714 / 3302 [*R*(int) = 0.0601]
Completeness to theta = 27.48	99.60%	99.20%	99.40%
Max. and min. transmission	0.9726 and 0.9564	0.9702 and 0.9648	0.9882 and 0.9504
Refinement method	Full-matrix least-squares on F^2		
Data / restraints / parameters	3249 / 0 / 195	3526 / 0 / 204	3302 / 0 / 263
Goodness-of-fit on F^2	1.063	1.038	0.778
Final R indices [*I* > 2sigma(*I*)]	R1 = 0.0470, wR2 = 0.1287	R1 = 0.0487, wR2 = 0.1314	R1 = 0.0402, wR2 = 0.0918
R indices (all data)	R1 = 0.0647, wR2 = 0.1389	R1 = 0.0797, wR2 = 0.1453	R1 = 0.0960, wR2 = 0.1238
Largest diff. peak and hole	0.194 and -0.232 e.$Å^{-3}$	0.262 and -0.152 e. $Å^{-3}$	0.268 and -0.214 e. $Å^{-3}$

Table S3-2. Crystal data and structure refinement for **4**, **5** and **6**.			
Identification code	**4**	**5**	**6**
Empirical formula	$C_{18} H_{19} N O_4$	$C_{18} H_{19} N O_3$	$C_{18} H_{19} N O_4$
Formula weight	313.34	297.34	313.34
Temperature (K)	293(2)	293(2)	293(2)
Wavelength (Å)	0.71073	0.71073	0.71073
Crystal system, space group	Monoclinic, P2(1)/c	Monoclinic, P2(1)/n	Monoclinic, P2(1)/c
Unit cell dimensions	a = 11.263(2) Å	a = 10.858(2) Å	a = 11.793(2) Å
	b = 12.388(3) Å	b = 13.738(3) Å	b = 11.504(2) Å
	c = 12.571(3) Å	c = 10.878(2) Å	c = 13.028(3) Å
	alpha = 90 deg.	alpha = 90 deg.	alpha = 90 deg.
	beta = 115.73(3) deg.	beta = 106.98(3) deg.	beta = 114.25(3) deg.
	gamma = 90 deg.	gamma = 90 deg.	gamma = 90 deg.
Volume ($Å^3$)	1580.1(6)	1552.0(5)	1611.7(6)
Z, Calculated density (mg/m^3)	4, 1.317	4, 1.273	4, 1.291
Absorption coefficient (mm^{-1})	0.093	0.087	0.091
F(000)	664	632	664
Crystal size, mm	0.40 × 0.35 × 0.32	0.61 × 0.22 × 0.20	0.45 × 0.34 × 0.11
Theta range for data collection	3.27 to 27.47 deg.	3.15 to 27.47 deg.	3.16 to 27.48 deg.
Limiting indices	-14<=h<=14, -16<=k<=16, -16<=l<=15	-13<=h<=14, -17<=k<=17, -14<=l<=13	-15<=h<=15, -13<=k<=14, -16<=l<=16
Reflections collected / unique	15209 / 3593 [*R*(int) = 0.0391]	14464 / 3520 [*R*(int) = 0.0732]	14918 / 3673 [*R*(int) = 0.0565]
Completeness to theta = 27.48	99.20%	99.10%	99.50%
Max. and min. transmission	0.9707 and 0.9641	0.9826 and 0.9488	0.9901 and 0.9603
Refinement method	Full-matrix least-squares on F^2		
Data / restraints / parameters	3593 / 0 / 213	3520 / 0 / 231	3673 / 0 / 208
Goodness-of-fit on F^2	1.054	1.025	1.064
Final R indices [I > 2sigma(I)]	R1 = 0.0470, wR2 = 0.1271	R1 = 0.0670, wR2 = 0.1429	R1 = 0.0614, wR2 = 0.1734
R indices (all data)	R1 = 0.0739, wR2 = 0.1391	R1 = 0.1489, wR2 = 0.1756	R1 = 0.1107, wR2 = 0.1981
Largest diff. peak and hole	0.194 and -0.179 e.$Å^{-3}$	0.154 and -0.167 e.$Å^{-3}$	0.271 and -0.243 e.$Å^{-3}$

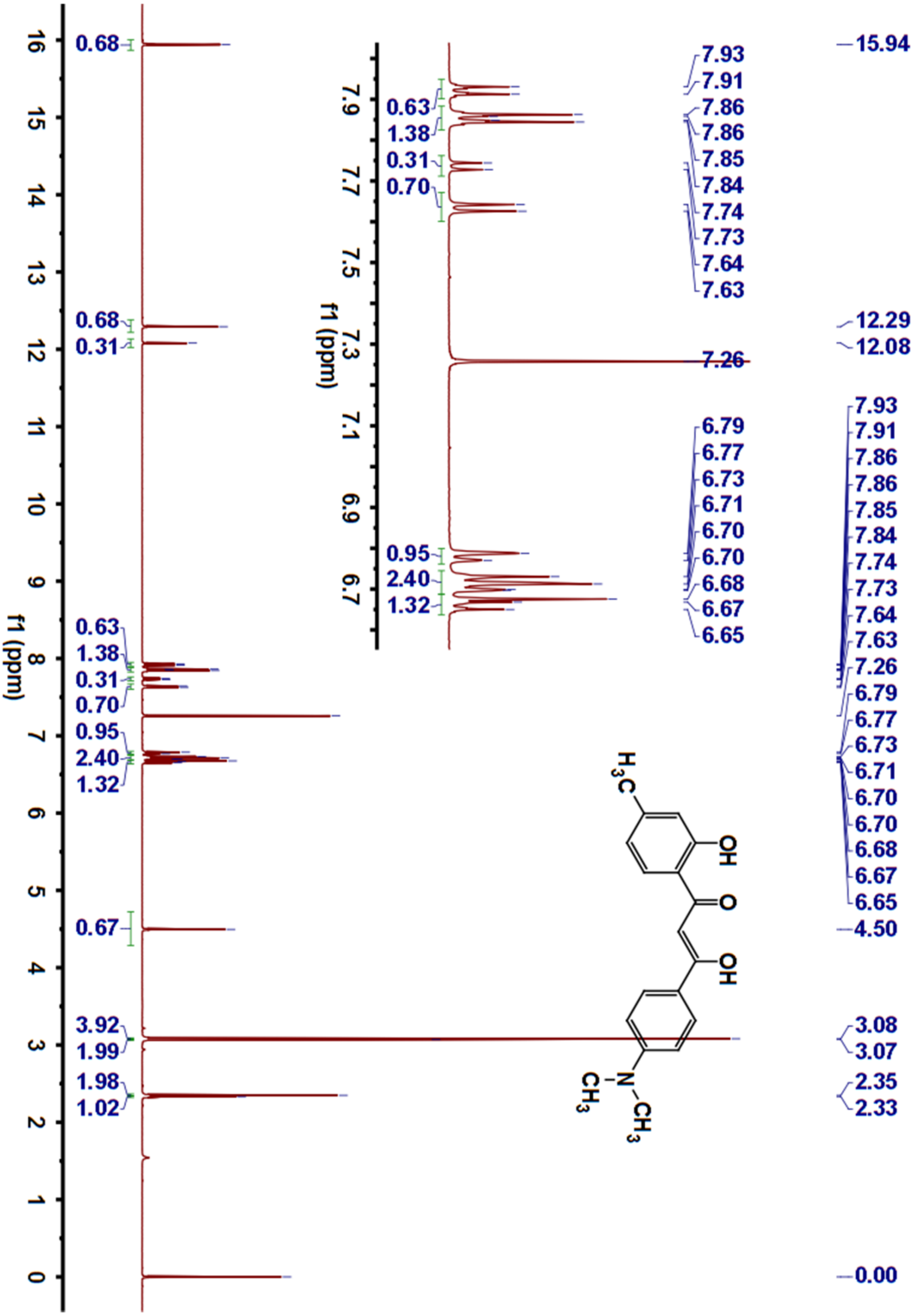

Figure S8. ^{1}H spectra of **2** recorded in $CDCl_3$ (500 MHz).

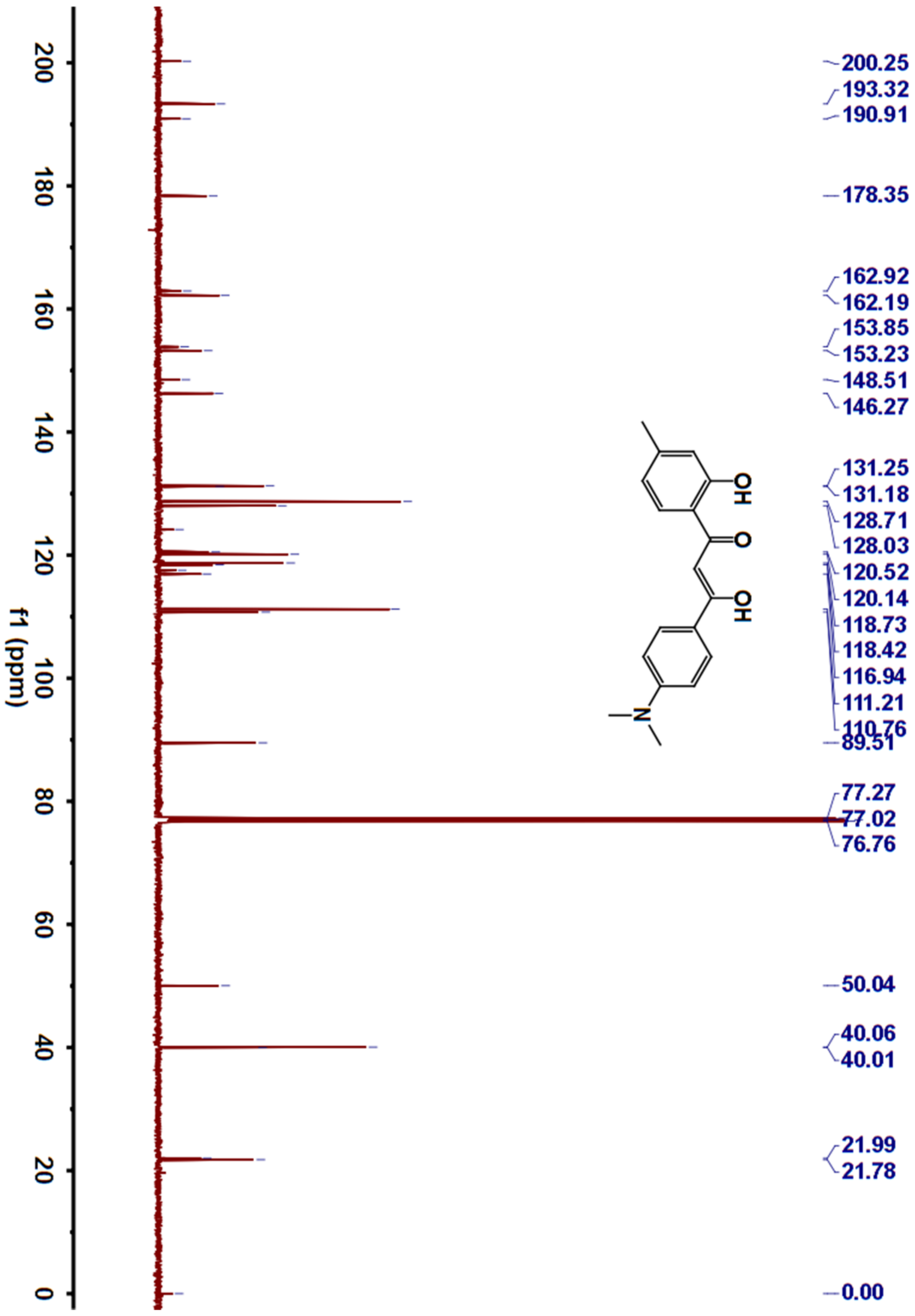

Figure S9. ^{13}C spectra of **2** recorded in $CDCl_3$ (125 MHz).

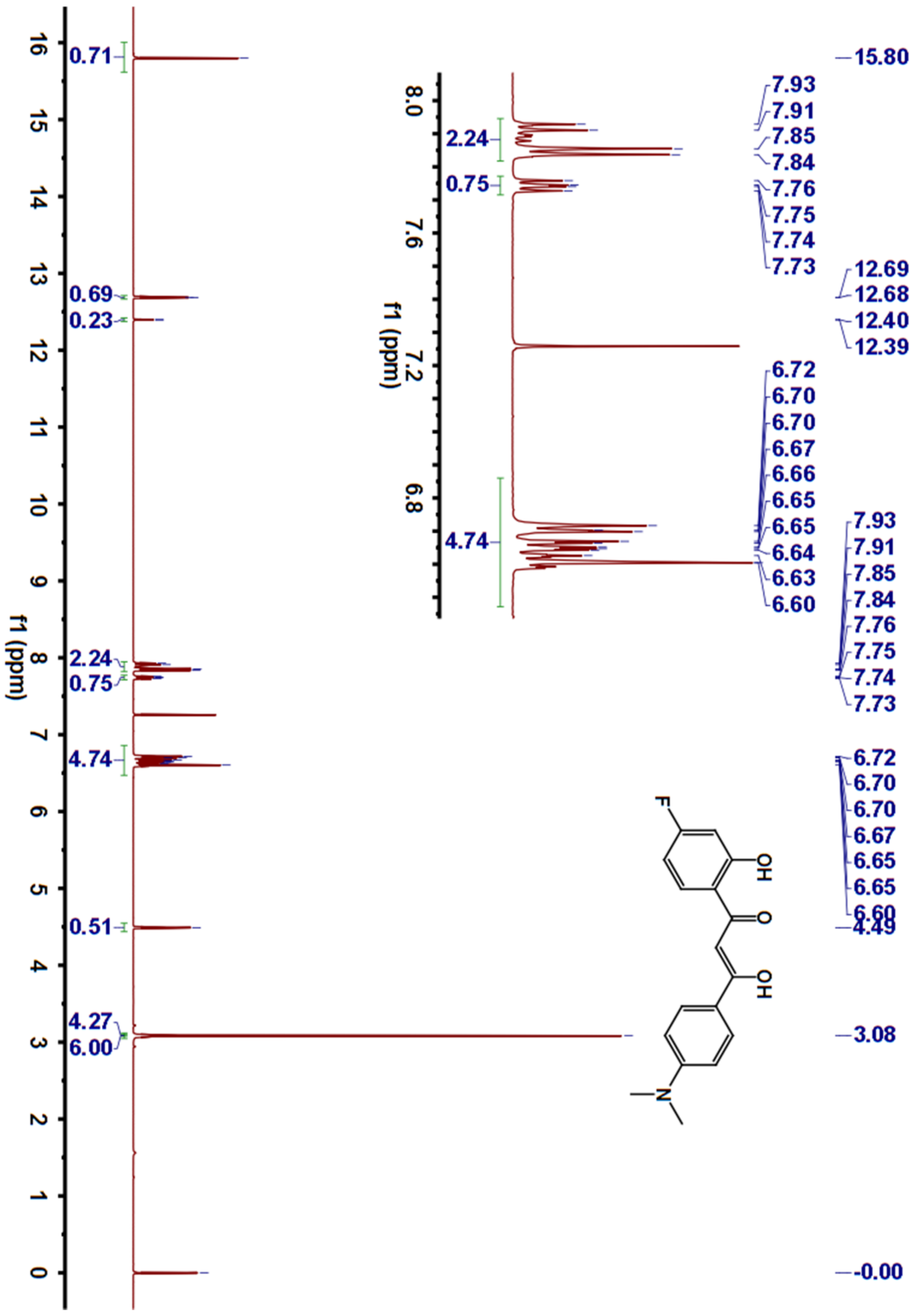

Figure S10. ^{1}H spectra of **3** recorded in $CDCl_3$ (500 MHz).

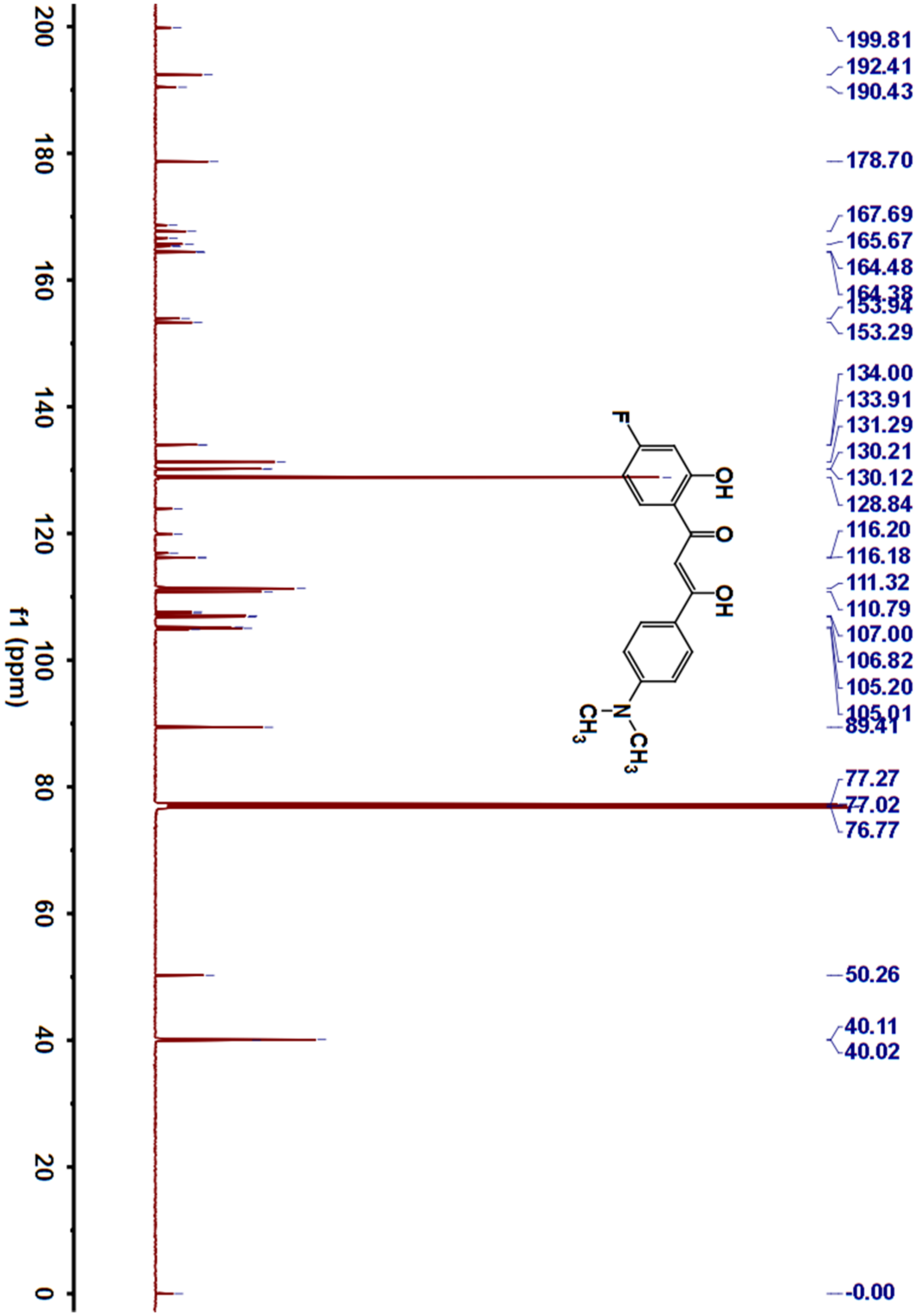

Figure S11. ^{13}C spectra of **3** recorded in $CDCl_3$ (125 MHz).

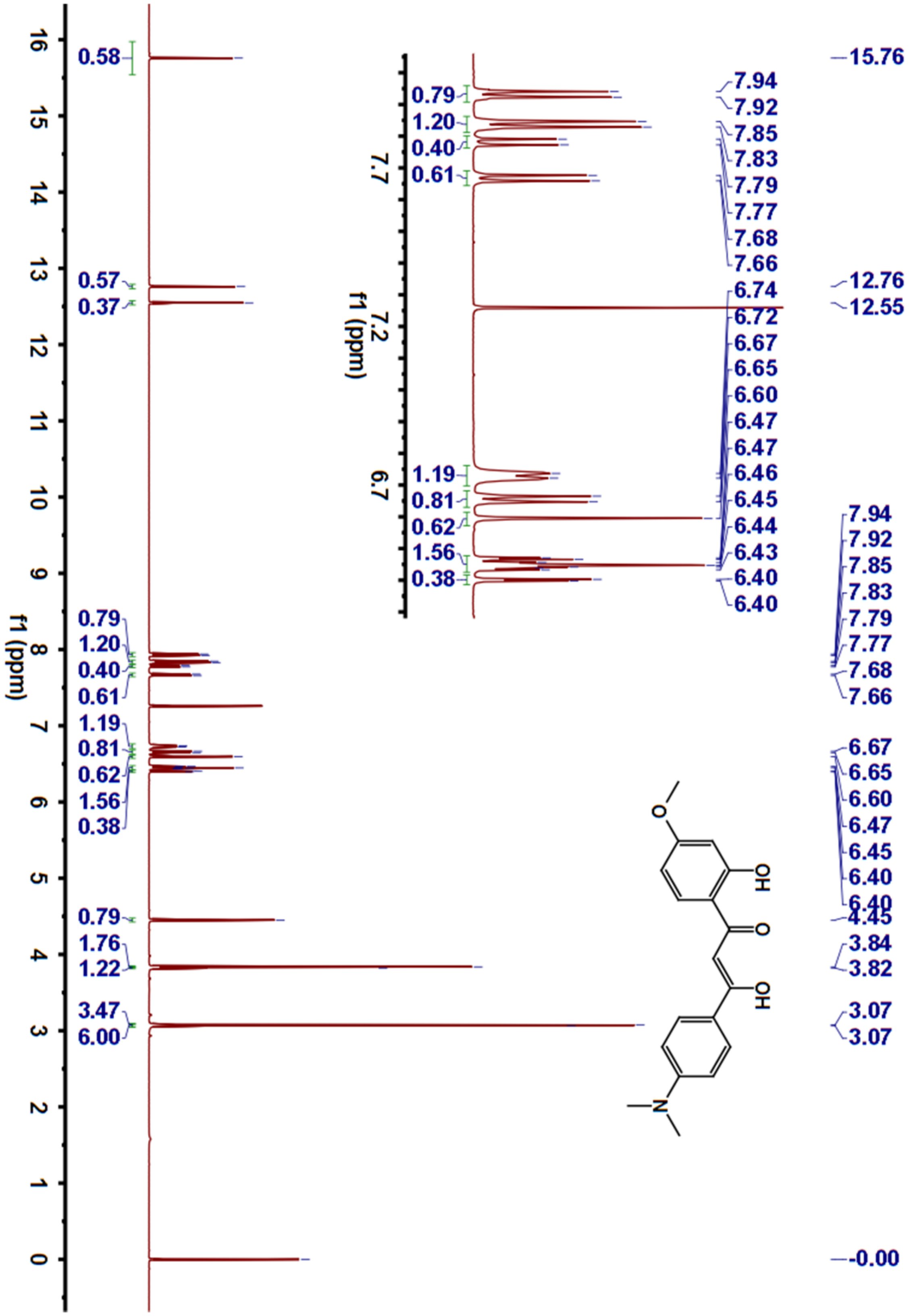

Figure S12. ^{1}H spectra of **4** recorded in $CDCl_3$ (500 MHz).

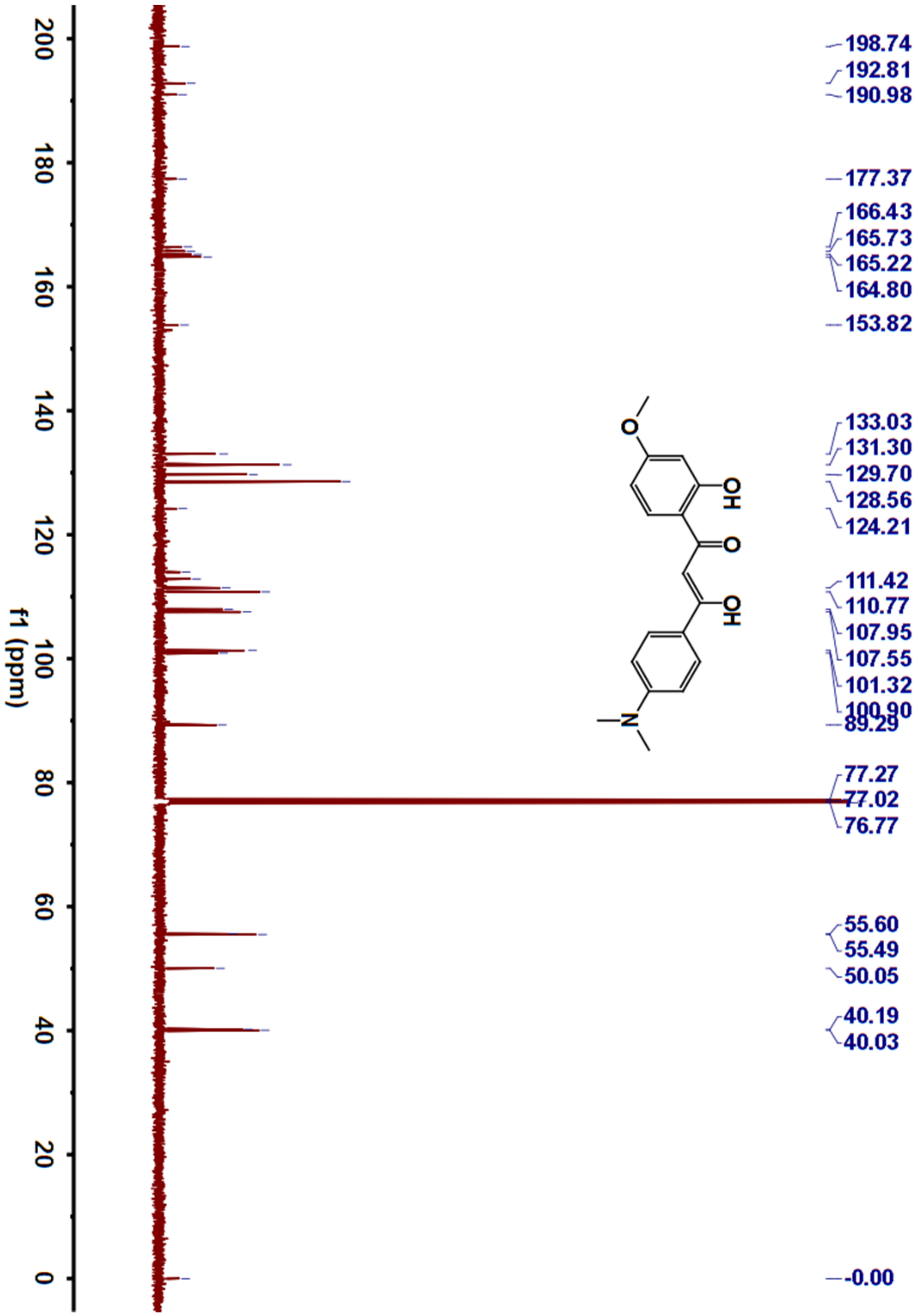

Figure S13. ^{13}C spectra of **4** recorded in $CDCl_3$ (125 MHz).

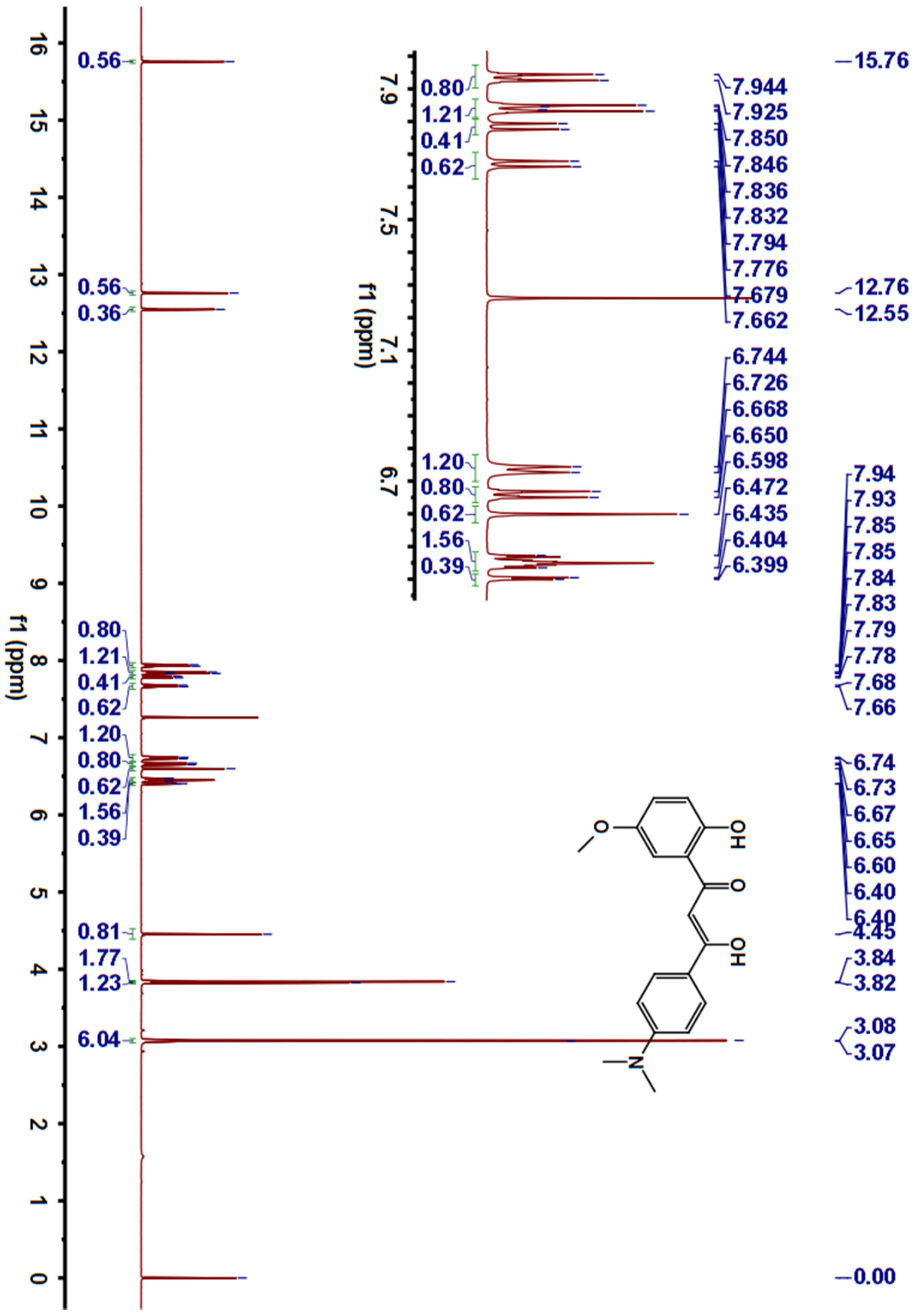

Figure S14. ^{1}H spectra of **6** recorded in $CDCl_3$ (500 MHz).

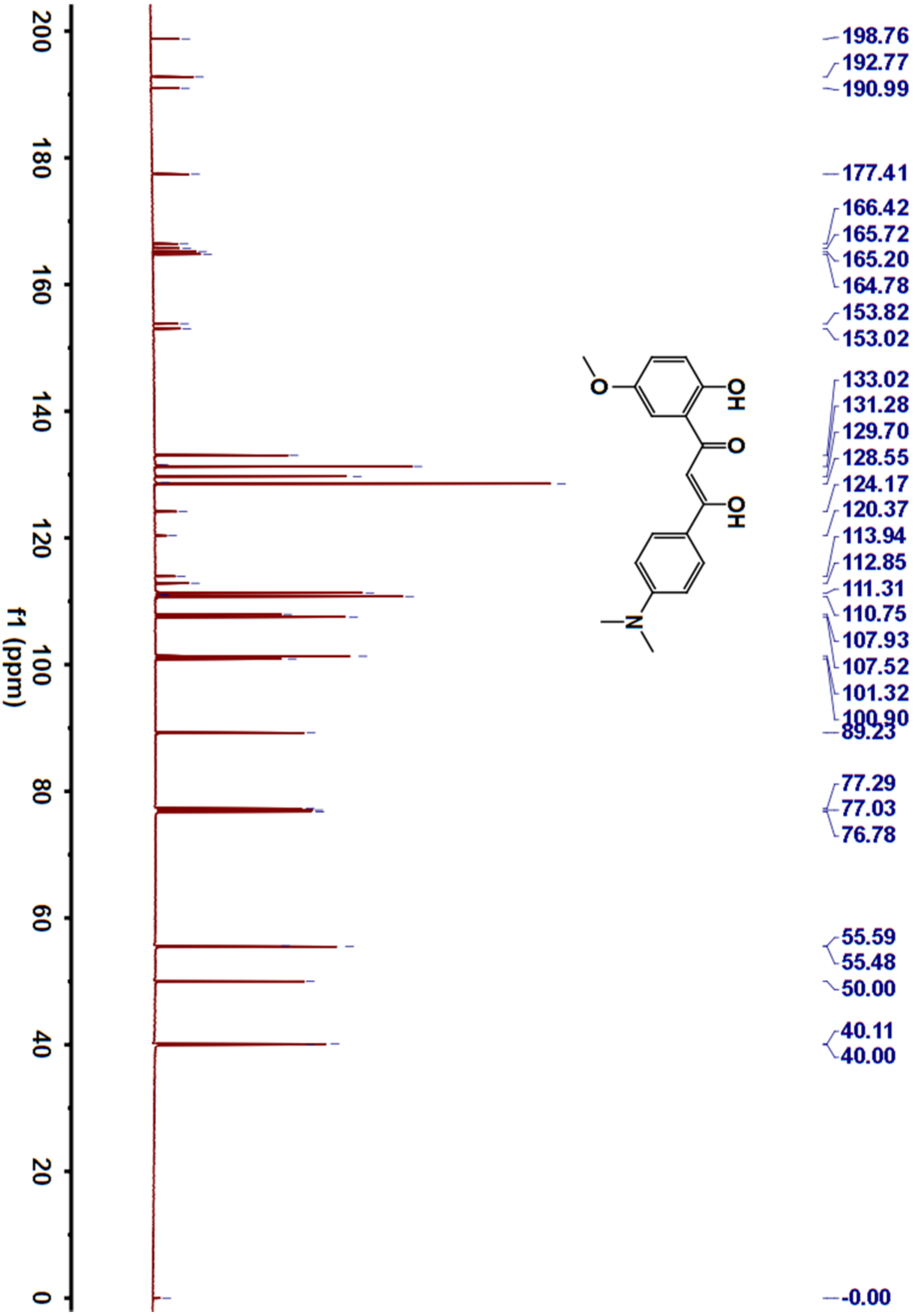

Figure S15. ^{13}C spectra of **6** recorded in $CDCl_3$ (125 MHz).
